# Molecular Modeling Insights into the Structure and Behavior of Integrins: A Review

**DOI:** 10.3390/cells12020324

**Published:** 2023-01-14

**Authors:** Igor Tvaroška, Stanislav Kozmon, Juraj Kóňa

**Affiliations:** 1 Institute of Chemistry, Slovak Academy of Sciences, Dúbravska cesta 9, 845 38 Bratislava, Slovakia; 2 Medical Vision o. z., Záhradnícka 4837/55, 821 08 Bratislava, Slovakia

**Keywords:** integrins, structure, mechanism, integrin ligand-interactions, cancer, inflammation, autoimmune disorders, antagonists, rational drug design

## Abstract

Integrins are heterodimeric glycoproteins crucial to the physiology and pathology of many biological functions. As adhesion molecules, they mediate immune cell trafficking, migration, and immunological synapse formation during inflammation and cancer. The recognition of the vital roles of integrins in various diseases revealed their therapeutic potential. Despite the great effort in the last thirty years, up to now, only seven integrin-based drugs have entered the market. Recent progress in deciphering integrin functions, signaling, and interactions with ligands, along with advancement in rational drug design strategies, provide an opportunity to exploit their therapeutic potential and discover novel agents. This review will discuss the molecular modeling methods used in determining integrins’ dynamic properties and in providing information toward understanding their properties and function at the atomic level. Then, we will survey the relevant contributions and the current understanding of integrin structure, activation, the binding of essential ligands, and the role of molecular modeling methods in the rational design of antagonists. We will emphasize the role played by molecular modeling methods in progress in these areas and the designing of integrin antagonists.

## 1. Introduction

Integrins, selectins, cadherins, immunoglobulins, and mucins comprise five major families of adhesion molecules [1,2]. These molecules mediate cell interactions in their environment and with the extracellular matrix. Some of these interactions are firm and stable; others are weak and short-lived and are vital for various physiological processes. Interactions of adhesion molecules are crucial in an adequately functioning the immune system, including leukocyte trafficking into tissue in healing processes [3,4] and finding and killing cancer cells [5]. However, they might also be involved in chronic and acute inflammatory diseases and promote cancer growth and metastasis [1]. Therefore, inhibitors of adhesion interactions have become potential therapeutics [1,6].

Integrins are a family of cell adhesion molecules that mediate cell–cell, cell–extracellular matrix, and cell–pathogen interactions. They fulfill vital roles in immune cell trafficking, migration, and immunological synapse formation during inflammation and cancer. Moreover, their interactions with ligands result in signal transduction pathways through a membrane [7]. Integrins are large transmembrane heterodimers made of two glycoproteins, called α and β subunits, non-covalently linked [8]. There are 18 α and 8 β subunits, which can theoretically assemble into 144 different heterodimers. However, until now, only 24 complexes have been identified and have functional and tissue specificity [9]. Integrins operate as complete receptors in the plasma membrane and bind to various cytoskeletal proteins and signaling molecules in the extracellular matrix. Notably, integrins are expressed on cell surfaces in an inactive conformation and are not able to bind ligands and transduce a signal. Their activity is regulated from inside the cells by a process called inside-out signaling [10,11,12]. Several diseases are associated with defects in integrins [13,14]. Therefore, it is unsurprising that integrins are targets for potential treatment in inflammatory diseases and cancer. Several reviews were published on various aspects of integrins, such as integrin structure and function [7,8,9,11,13,15,16,17,18,19,20,21,22], integrins as therapeutic targets [14,20,23,24,25,26,27,28], and integrins in functional biomaterials [29,30,31]. Therefore, this is not intended to be an exhaustive review of all structural and functional studies on integrins. We want to give the reader an overview of how several molecular modeling methods contributed to shedding some light on the particular features of integrins, such as their structure, conformational behavior, and activation. We will also address the use of molecular modeling methods in the design of ligands and an estimate of their activity and selectivity.

## 2. Computational Modeling Methods

Proteins perform an enormous diversity of biological functions associated with their naturally evolved three-dimensional (3D) structures, determined by genetically encoded amino acid sequences. Proteins exist as an ensemble of conformations in a dynamic equilibrium, depending on their biological environment, which influences their functions. From both the experimental and computational points of view, understanding proteins’ dynamic behavior and the characterization of their structural features have been challenging for decades. Experimental data provide information about a single molecule’s properties or ensemble average values. Computational methods provide information on the distribution in the ensemble at the atomic level. Thus, the combinations of experimental and molecular modeling methods provide a unique way to solve this demanding task. It is beyond the scope of this review to give a detailed description of all used computational methods, and in the following chapter, only a brief overview is presented.

In the past decades, considerable increases in computing power and several emerging computational methods have provided tools for describing 3D structures and properties of biomolecules, with potentially wide-ranging applications in biology, medicine, pharmacology, biotechnology, and the design of new materials. Current computational approaches span wide-ranging methods from ab initio quantum mechanics (QM) to coarse-grained methods. These methods are combined with existing algorithms that scan configurational space, such as deterministic molecular dynamic simulation (MD), heuristic Monte Carlo method (MC), or enhanced sampling techniques. The choice of the most appropriate method depends on the complexity of the studied system, the details needed for understanding the studied properties or chemical/biochemical processes, and computational resources.

### 2.1. Quantum Chemistry Methods

Until recently, molecular orbital methods, also known as the self-consistent field (SCF) approximation, were used in QM computations of biomolecules [32,33]. The accuracy of ab initio QM calculations is mainly affected by the quality of the atomic orbitals used to build the molecular orbitals and the inclusion of electron-correlation effects [34]. Although various methods were developed to include electron correlation, they require a colossal computer effort. Therefore, QM ab initio calculations of the structure and behavior of large systems are restricted. During the past two decades, the density functional theory (DFT) method [35] has become the method of choice for investigations of biomolecular systems due to its satisfactory accuracy and lower computational resources compared to QM ab initio methods. The DFT method describes molecules using the electron density instead of the wave function used in the QM ab initio methods. The reliable exchange-correlation functional is crucial for proper DFT calculations, and its quality is constantly improving [36,37,38]. In particular, the B3LYP functional with the 6-31 + G* basis set became the most popular functional for calculating conformational sampling of medium-size molecules. Recently, several new functionals, including M05-2X, M06-2X, MPW1K, and PWB6K, were developed that reasonably well predict the structure of large biomolecules [39,40]. Then, conformational equilibrium of the final set of conformers is usually based on calculated energies performed using the 6-311 + +G** basis set. Despite the considerable progress in QM calculations, the applications of good quality QM methods are limited to relatively small biological systems with a number of atoms ~300, e.g., the active site of enzymes, or the binding sites of proteins. However, in processes where bond-breaking and bond-forming occur, QM methods are not avoidable. To solve this limitation of QM methods, the combined quantum mechanics–molecular mechanics (QM/MM) approach was proposed [41] and soon became very popular for calculating the enzymatic reaction. In QM/MM methods, the relevant part of the system, such as the active site of an enzyme, is calculated at the electronic level with QM methods. In contrast, the remaining portion of the system is calculated at the atomic level using MM methods. The development and application of QM/MM methods have been presented and discussed in several recent reviews, which readers should refer to for further details [42,43,44,45,46,47,48].

The defragmentation methodology is another way to deal with large protein systems at the QM level [49]. The fragment molecular orbital (FMO) method [50,51] is one such approach. The pair interaction energy decomposition analysis (PIEDA) with the FMO method was recently used to analyze interaction energies in different biomolecular systems [52,53,54,55,56,57,58].

### 2.2. Molecular Mechanics (Force Fields) Methods

A cheaper alternative to expensive QM calculations of the energy of a given biomolecular system are molecular mechanics calculations based on the laws of classical mechanics. Molecular Mechanics (MM) or Force Field (FF) methods consider atoms in molecules as charged spheres linked by springs of different elasticity. MM methods use classical potential functions to calculate a molecule’s structure and potential energy in a particular conformation. These equations, together with the set of parameters (force constants, equilibrium values, and atomic charges), determined using structural and thermodynamic experimental or QM data, are called force fields. The potential energy of a molecule is the function of the position of all atoms. Generally, the potential energy is expressed as the sum of individual functions for bond lengths stretching, bond angles bending, torsional angle energy, electrostatic, non-bonded, and dispersion interactions. It is noteworthy that total energy has no absolute meaning. It serves only as a comparison of different conformations of a particular molecule. MM’s main advantages are considerably lower computing power and CPU time requirements than QM. In the last decade, force fields have been continuously improved [59,60,61,62,63]. Nowadays, the developed force fields such as AMBER [64], CHARMM [60], GROMOS [65], and OPLS [66] provide tools that can address questions related to a protein 3D structure and characterize its conformational ensemble.

During many biochemical processes, the charge distribution on atoms usually changes. However, MM calculations calculate electrostatic contributions to a system’s potential energy with fixed atomic charges. Therefore, in the last decade, several attempts were focused on developing general polarizable force fields for biochemical simulations [63,67]. Of the different methods used to account for polarization in classical MM, the Drude oscillator model [68] is the most popular and is included in various software suits [69]. In the last two decades, considerable progress has been made in developing polarizable force fields and their application to biochemical systems [70]. Though various improved results were obtained, some challenges remain to be solved [63]. Additionally, their general applications are hampered by computationally expensive requirements for calculations of large systems.

Applications of MM methods to chemical reactions are impossible due to the predefined bonding topology, which cannot describe processes when bonds are broken and formed in a chemical reaction. Recently, the ReaxFF method was developed [71,72]. Four force fields of ReaxFF have been parameterized for biochemical systems [73,74,75,76] and employed for molecular dynamics (MD) simulations. Force fields were established using different training sets and algorithms used for the parameterization. Though the force field was not parameterized for glycosyltransferases [73], its validation on a real glycosyltransferase ppGalNAT2 led to a reasonable description of the enzymatic reaction comparable to QM/MM DFT calculations [77]. Although the parametrized force field is far from being final, the obtained results are encouraging, suggesting that ReaxFF has the potential to describe enzymatic reactions with accuracy similar to QM/MM DFT with a computational cost of 4–6 orders of magnitude lower. The performance of developed ReaxFF force fields was recently evaluated [78]. It showed that though they perform well for specific applications, they are mostly not transferable to general applications involving amino acids.

Coarse-grained models were developed to speed up simulations of large systems by grouping several atoms into a single particle-bead [79]. Various schemes were used to define beads [80]. The most common is to form one bead from four non-hydrogen atoms. This considerably decreases the system’s dimensionality, accelerating calculation by several orders of magnitude compared to classical MM calculations. Moreover, “bonds” between beads vibrate with lower frequencies, and, as a result, a larger simulation step can be used. On the other hand, lower dimensionality does not allow a proper evaluation of some thermodynamic properties, e.g., entropy. Of course, coarse-grained force fields must be developed for biomolecules by using equations describing interactions analogous to those in classical MM force fields. The Martini force field is the most popular model developed for various biochemical systems [81,82,83]. The coarse-grained models are instrumental in describing systems dominated by macroscopic properties. However, they are inappropriate for phenomena where atomic (detailed) interactions are crucial [84].

### 2.3. Molecular Dynamics Simulations

Biomolecules are flexible structures that exist as a dynamic ensemble of conformations with equilibrium depending on their free energy surface, a function of a molecular structure. The topology of these high-dimensional surfaces is very complex, with many local minima connected by pathways via barriers due to a vast number of degrees of freedom and depending on the biological environment. Determining the molecular structure by directly applying the above-discussed computational methods using geometry optimization procedures provides a single structure. Usually, it leads to the nearest local minimum from the starting structure on the energy surface. Since experiments generally provide the ensemble average values, reliable calculations must consider the most relevant structures in dynamic ensemble. In other words, calculations must sufficiently sample a particular biomolecular system’s configuration space. The two most common techniques that scan configuration space and provide reasonable ensemble averages are Monte Carlo (MC) and molecular dynamics (MD) simulations. These methods can be combined with the energy calculated by QM, QM/MM, or MM FF methods.

The MC and MD simulations can determine structures or refine structures from experimental data and characterize a system’s thermodynamic or other parameters at equilibrium. In both cases, the adequate sampling of the configuration space is essential for obtaining the correct Boltzmann-weighted ensemble. To examine the actual dynamics of the biomolecular system, where the changes in the structure and their changes over time are of primary interest, only MD can provide the necessary information [85]. In MD simulation, conformational sampling is determined using the Newtonian equation of motion applied to the potential energy function of the molecular system [86,87]. Given a starting set of atomic positions and velocities, the force acting on each atom is calculated by taking the potential energy gradient. A tiny step forward in time is required (typically of the order of a few femtoseconds) to achieve energy conservation. New positions and velocities are calculated by integrating Newton’s equation of motion using the time-step size and the old positions, velocities, and accelerations. The quality of the method for calculating energy determines whether MD simulations provide a sufficient sampling of the energy surface, whether sampled conformations are realistic, and whether the obtained evolution of molecules over time is credible.

### 2.4. Enhanced Sampling Algorithms

The challenge is that high-energy barriers separate different conformations and transitioning between them requires very long simulations on a multi-dimensional hilly free energy surface. Sampling biologically relevant time scales (milliseconds) with femtosecond steps requires more than a trillion integration time steps and calculations of interactions between tens of thousands of atoms at each step. Though the enormous progress in computational resources permits increasing simulation time to the millisecond time scale for millions of atoms [88], such simulations of biological systems are far from routine techniques and even require specialized supercomputers. A straightforward approach to accelerate the thermodynamics calculation is to lower the energy barriers on the energy surface, thus increasing the sampling transition regions. Intuitively, this can be achieved by increasing the system’s temperature or by adding bias potential to the system’s energy. Recently, several enhanced sampling methods have emerged that accelerate the dynamics of such systems. The enhanced sampling methods, such as umbrella sampling [89], replica exchange molecular dynamics (REMD) [90,91], metadynamics (MTD) [92,93], variationally enhanced sampling [94], and integrated tempering sampling [95,96], belong among the widely used.

Interpreting configurational ensembles from MD simulations data and efficient conformational sampling on a high-dimensional energy surface requires reducing the studied problem’s dimensionality. The dimensionality reduction provides structural coordinate(s) called collective variables (CVs). The choice of CV is crucial for designing simulations. The values of CVs should clearly distinguish between different conformations of the studied system, should be calculated as a function of atomic coordinates, and their number should be limited [97]. Simple CVs that meet these conditions represent stereochemical parameters, such as atom–atom distances, bond and dihedral angles [92], a radius of gyration, coordination number, ring-puckering coordinates [98], or pharmacophore descriptors [99], etc. In addition, their combination can be appropriate in some cases. Many enhanced techniques are included in biomolecular software packages, such as AMBER [100], GROMACS [101], and NAMD [102,103]. The enhanced sampling approaches were recently reviewed [84,104,105,106,107].

### 2.5. Protein Structure Prediction

Complications in the cloning, expression, and purification of milligram quantities of the protein that affects obtaining a sufficient amount of material and difficulties associated with crystallization often hinder the experimental elucidation of a protein structure. In this context, it is not surprising that the development of computational methods predicting protein structure has gained much interest [108,109]. Various computational methods such as homology modeling (also known as comparative modeling), fold recognition and threading, and first principles (ab initio or de novo) techniques with or without database information were used for protein structure prediction. 

A homology model of the given protein (target) is constructed from its amino acid sequence and an experimental three-dimensional structure of related homologous proteins (templates), based on the assumption that proteins with sequence similarity also have structural similarity [110]. Usually, homology modeling proceeds with these main steps: the identification of related sequences of known structure; the alignment of the target sequence to the template structures; the modeling of structurally conserved regions using the known templates; modeling side chains and loops that are different than the templates; and finally, refining and evaluating the quality of the model through conformational sampling by MD simulations. The degree of sequence similarity and the accuracy of template models are decisive factors in the quality of the homology model. Widely used programs for predicting the 3D structure of proteins are MODELLER [111], Prime [112,113], and an Automated Comparative Protein Modelling Server SWISS-MODEL [114].

It is often impossible to find a protein with identity in a pair-wise alignment between target and template proteins higher than 25%. In this case, the results of homology modeling are unreliable. Instead, protein threading, also known as fold recognition [115], can be used for protein modeling. The prediction is made by placing (threading) each amino acid in the target sequence to a position in the template structure and evaluating how well the target fits the template. After the best-fit template is selected, the structural model of the sequence is built based on the alignment with the chosen template. Threading works by using statistical knowledge of the relationship between the structures deposited in the PDB and the protein sequence one wishes to model. TREADER [116] and RaptorX [117] represents software developed for this method’s application.

The structure prediction for proteins lacking structural similarity to a protein in the protein database is highly challenging and requires extensive computer resources. The prediction of protein 3D structures based solely on their primary structure attracted the interest of many computational labs for many years, and several ab initio (de novo) approaches were developed [108,109,118]. Ab initio methods require accurate energy functions that correctly describe the location and orientation of amino acid side chains, as well as their residue–residue interactions, and can be used for the final refinement to provide a high-resolution structure, an efficient conformational sampling strategy, and ranking criteria for a choice of near-native models from an ensemble of models. Despite considerable progress in developing ab initio algorithms, no approach has been able to reliably produce models with atomic accuracy up to now.

The breakthrough came in the last year. Two groups have independently developed the deep-learning-based methods AlphaFold2 [119,120] and RoseTTAfold [121]. AlphaFold2, developed by DeepMind company, is an artificial intelligence system that predicts the 3D structure of a protein from the primary structure with accuracy comparable with experiments. Simultaneously, the academic team developed RoseTTAfold, producing similar results [122]. These novel machine-learning approaches incorporate physical and biological knowledge about protein structure to design deep-learning algorithms. A collaboration between the European Molecular Biology Laboratory’s-European Bioinformatics Institute (EMBL-EBI) and DeepMind has predicted structures for over 200 million proteins that are freely available at the AlphaFold Protein Structure Database (the FTP site: https://ftp.ebi.ac.uk/pub/databases/alphafold (accessed on 6 December 2022)).

### 2.6. Molecular Docking

Ligand binding is a key process in various biological processes and drug design. Therefore, a detailed description of interactions and prediction binding affinity between macromolecular receptors (proteins/DNA) and small molecules (ligands) is essential for a rational drug design and discovery. Today, a variety of docking algorithms are available [123,124,125,126].

**Figure 1:**
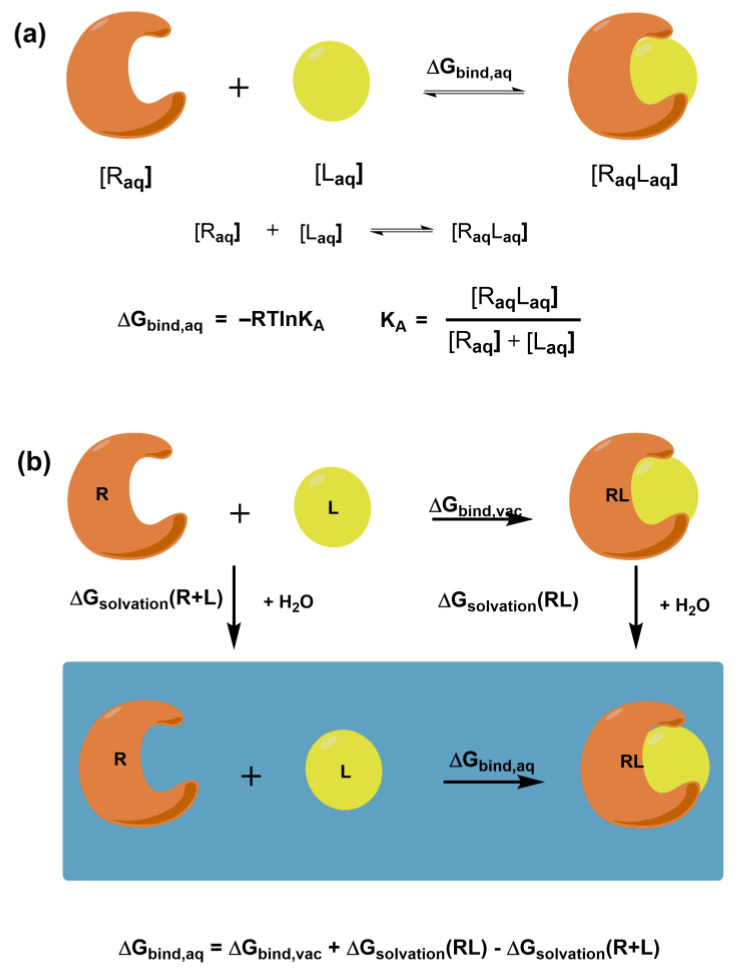
Schematic representation of (**a**) the free energy of binding ΔG_bind,aq_ for the receptor-ligand complex and its relation to binding affinity; (**b**) the thermodynamic cycle for calculating the free binding energy between the receptor and ligand.

The ultimate goal of molecular docking methods is to correctly predict the ligand’s most favorable orientation and position (pose) at the binding site of the target macromolecule. The docking procedure generates multiple conformations, while exploring a whole conformational space is crucial. The methods also estimate the receptor-ligand binding free energy, often using the so-called scoring function. The free energy of binding ΔG_bind,aq_ characterizes the strength of the interaction between a macromolecular receptor and a particular ligand under equilibrium (Figure 1a) and binding affinity. It is noteworthy that knowledge of K_A_ is not necessary to predict the correct complex structure. However, in the case of inhibitors, prediction of their potency is crucial. Intermolecular electrostatic, non-bonded, and hydrogen bonding interactions between receptor and ligand and intramolecular structural changes in both molecules determine the magnitude of ΔG_bind,aq_. They all contribute to the binding enthalpy. A desolvation and a loss in rotational and translational degrees of freedom contribute to the binding entropy. Figure 1b shows the thermodynamic cycle for a macromolecular receptor and a ligand in the aqueous solution and vacuum that can be used to calculate ΔG_bind,aq_ in solution.

Docking methods require knowledge of the receptor’s 3D structure. Generally, the receptor coordinates are obtained from solved X-ray or NMR structures. If they are absent, the predicted protein models can also be used [125]. Atomic, surface, and grid representations of receptors are used for docking. A successful docking procedure requires an accurate and efficient sampling of the ligand and receptor flexibility. Various algorithms are used to treat ligand flexibility, such as systematic methods using the conformational search or incremental construction, e.g., in programs DOCK [127], FlexX [128], and Glide [129]; random or stochastic methods using MC or genetic algorithm, e.g., in programs Gold and autoDock [130]; and simulation methods using MD or MTD simulations, e.g., in programs DOCK, autoDock, and Glide. Treating receptor flexibility requires considerable computational time; therefore, a receptor is usually kept rigid. Some programs execute so-called “soft docking” sampling of the conformational space of relevant side chains in the binding site. Docking approaches can be combined with different computational methods for ranking predicted poses. The crucial need is to correctly predict the binding conformation of a ligand and distinguish between correct poses and false ones. Generally, three groups of scoring functions are used: force-field-based, empirical-based, and knowledge-based [125]. Dynamic simulations using MD and enhanced simulation techniques have become possible for molecular docking [126]. They consider the complete structural flexibility of both a ligand and receptor. Recently, well-tempered metadynamics was successfully applied to design an inhibitor of the αvβ3 integrin [131]. Though these methods are instrumental in providing quantitative values of the free energy and kinetics of binding, they are too computationally expensive for routine calculations in high-throughput screening. 

## 3. Structure of Integrins

**Figure 2:**
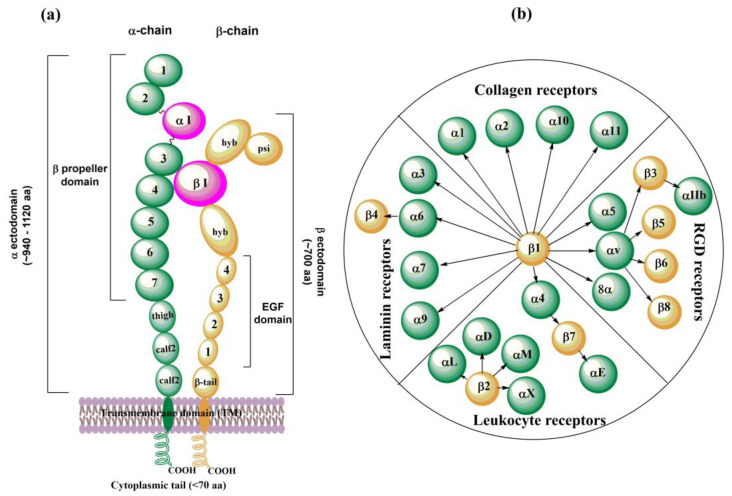
Schematic representation of (**a**) an arrangement of domains in the α- and β-subunit of integrins; (**b**) 24 distinct integrins divided into subfamilies according to their ligand specificities, adapted from ref. [8,15].

Integrins are membrane glycoproteins composed of α and β subunits that form a heterodimer. Both subunits consist of well-defined domains: a large extracellular domain (ectodomain) and a relatively short transmembrane domain with ~60 amino acids (aa). The exception is the β4 integrin [132] with ~1000 aa and cytoplasmic domain [20] (Figure 2a). The integrin cytoplasmic domain modulates crucial cell processes by interacting with various skeletal proteins and intracellular signaling molecules [9]. Two subunits in integrin complexes are held together by non-covalent bonds and form a ligand-binding site on the top of the two subunits. The ectodomain of α-chain is larger than that of β-chain: ~ 940𠀓1120 aa vs. ~700 aa. An α subunit ectodomain consists of two calf domains, a tight, and a seven-bladed β-propeller. The β subunit consists of a β-tail domain, four epidermal growth factor (EGF) modules, a hybrid domain with the inserted βI domain, and a plexin-semaphorin-integrin (PSI) domain.

There are 18 different found α subunits (α1–α11, αv, αIIb, αD, αL, αM, αX, and αE) and eight found β subunits (β1–β8). Nine of eighteen α subunits, namely α1, α2, α10, α11, αD, αL, αM, αX, and αE, have inserted the αI domain between the second and third blade of the β-propeller, which is crucial for the formation of a ligand binding region. This region also contains a Metal Ion-Dependent Adhesion Site (MIDAS) containing divalent cations such as Mg^2+^, Ca^2+^, or Mn^2+^. In the other nine α subunits (α3–α9, αv, and αIIb), the αI domain is missing, and a βI domain from an α-propeller domain in the α subunit headpiece and the MIDAS in the β subunit are responsible for forming the ligand binding region. In this case, other metal ion sites were also found similar to βI MIDAS; of the two ADMIDAS (Adjacent to MIDAS) sites, one of them is called a synergistic metal ion-binding site (SYMBS). Twenty-four integrins were identified in humans and can be classified according to their ligand-binding properties (Figure 2b) or tissue expression [8,15].

### 3.1. Glycosylation of Integrins

**Figure 3:**
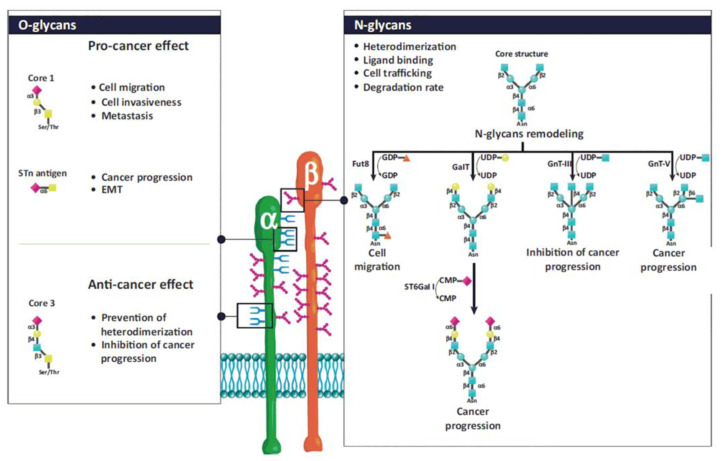
Schematic representation of *N*- and *O*-glycosylation of integrins by glycosyltransferases and associated functions. The *N*-glycan structure is involved in heterodimerization, ligand binding, cell trafficking, and the degradation rate of integrins. *N*-glycans regulate cell adhesion and migration and, consequently, cancer progression. Fut 8, α1,6-fucosyltransferase; GalT, hydroxyproline-O-galactosyltransferase; GnT-III, β1,4-N-acetylglucosaminyltransferase III; GnT-V, β1,6 N-acetylglucosaminyltransferase V; ST6Gal-I, ST6 β-galactoside α2,6-sialyltransferase I. Reprinted with permission from reference [135].

Glycan structures added to integrins by post-translational modifications contribute to their structural and functional diversity [133,134,135,136,137,138,139]. The glycosylation of proteins is a step-wise process carried out by glycosyltransferases. Glycosyltransferases (GTs) catalyze the transfer of glycosyl residue from a donor to an acceptor molecule [48]. The *N*- and *O*-glycosylations are the most frequent types of glycosylation. There are sufficient data linking aberrant glycosylation with pathological conditions, including chronic inflammation, immune diseases, cancer progression, and metastasis [48,140,141,142,143]. *N*-glycans presence is crucial for the association of both subunits into heterodimers, their stability, conformation, and interactions with ligands. For example, α5β1 and α3β1 integrins contain 14 and 12 *N*-glycosylation sites on α and β subunits, respectively. Their presence is crucial for interactions with fibronectin and laminin, mediating cell adhesion, migration, differentiation, and apoptosis [134]. However, from multiple *N*-glycosylation sites, only those located on specific motifs have these roles [144,145]. Integrins also contain *O*-glycans associated with the adhesion and migration of tumor cells, but their functions are less investigated due to difficulties in their isolation. Details about the influence of particular glycan structures and GTs responsible for their biosynthesis can be found in references [133,134,135,138] and are illustrated in Figure 3.

### 3.2. 3D structures of Integrins

X-ray crystallography, NMR spectroscopy, cryogenic electron microscopy, and molecular modeling methods solved the integrin structures and contributed to understanding their behavior. The first 3D structure of an integrin was the crystal structure of the integrin ectodomain for αvβ3 [146]. Up to now, there are more than 100 solved structures concerning various integrins’ parts, usually in complex with different ligands. The solved integrin-ligand complexes revealed the structures of binding sites and crucial interactions between inhibitor and ligand [20]. It is beyond the scope of this paper to discuss all X-ray structures. Readers can find relevant information in reviews on this subject [7,13,20,147,148,149]. In this section, we will discuss some solved structures of integrins, as well predicted 3D structures by homology modeling.

**Figure 4:**
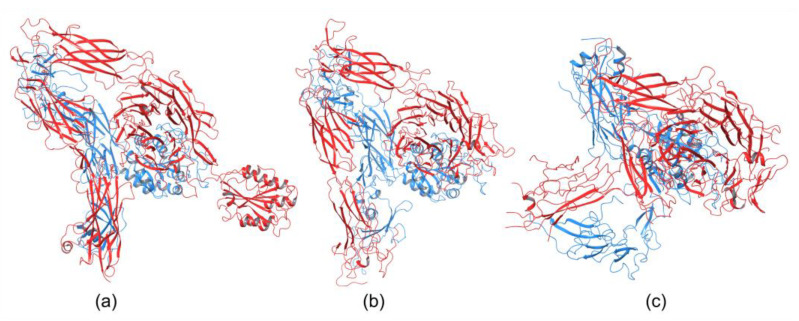
Crystal structures of integrin (**a**) containing αI domain (4NEH) in metastable state and integrins lacking the αI domain (**b**) αvβ3 (3IJE) and (**c**) αIIbβ3 (4CAK) in the bent conformation.

Integrins play an essential role in the immune system by mediating leukocyte adhesion and their transmigration from blood to tissue during leucocyte adhesion [150]. Therefore, it is unsurprising that integrins involved in immunological functions were studied more intensively than others. The αXβ2 integrin was the first solved structure of the ectodomain containing the αI domain [151]. The integrins αvβ3 and αIIbβ3 belong to the most investigated. These integrins are present on platelets and are associated with platelet functions in hemostasis and thrombosis, and they also participate in cancer progression [152]. The crystal structures of the complete integrin αvβ3 ectodomain plus α/β transmembrane fragment [153] and the intact integrin αIIbβ3 in a nanodisc lipid bilayer were solved recently [154]. Both integrins adopted a similar bent conformation, in which the ligand binding site is near the membrane surface. The crystal structures of an αI-containing αXβ2 (PDB entry 4NEH) and αI-lacking integrins αvβ3 (PDB file 3IJE) and αIIbβ3 (4CAK) are shown in Figure 4.

**Figure 5:**
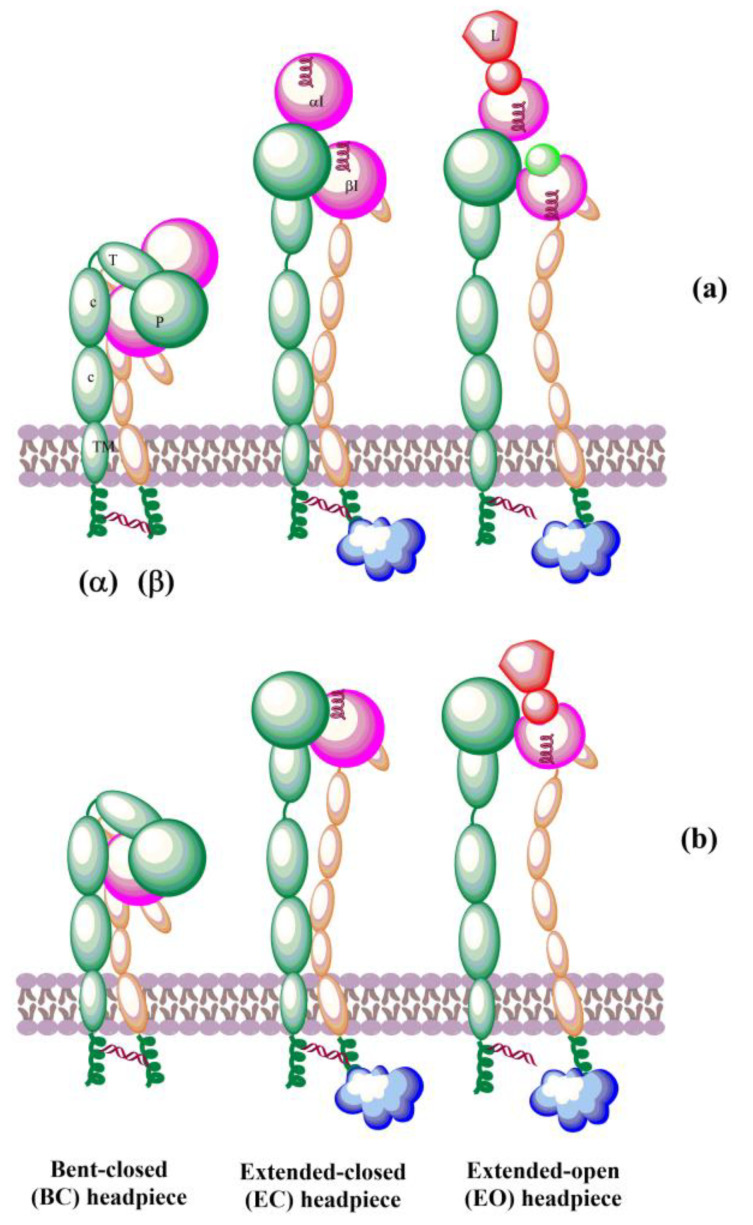
Schematic representation of domain architecture during activation of integrins that (**a**) contain or (**b**) lack an αI domain. L = ligand.

The solved crystal structures of integrin ectodomains and I domains [7,20,21,149,155] revealed that integrins exist during activation in the dynamic equilibrium of at least three major conformers: bent-closed (BC), open-closed (OC), and open-extended (OE)] [7,156,157]. Three conformers are schematically shown in Figure 5. Interactions of integrins with extracellular and cytosolic ligands (activators) trigger a large conformational movement that changes conformational equilibrium. In the absence of a ligand, a salt bridge interaction between helices of the cytoplasmic tails of α and β subunits hold the resting integrin in a low-affinity conformation [158]. Interactions of some protein activators, e.g., talin, with CT of β-subunits and membrane break this salt bridge, separate the α- and β-subunits, and the integrins switch to an extended conformation [159] of the α and β ectodomains that retains its low ligand affinity. Then, integrins interacting with extracellular ligands change to an open-extended, high-activity conformation [160]. It was observed [161] that after activation, integrins form ~100 nm clusters of ~50 integrins assisted in an early adhesion of cells. 

Recently, the conformational equilibriums of three conformers of the α5β1 integrin have been investigated by kinetics measurements using three different ligands [156]. The determined values of the free energy ΔG for the bent-closed (BC) and the extended-closed (EC) conformer are in the range from −1.2 kcal/mol to −1.8 kcal/mol and −0.7 kcal/mol to −1.2 kcal/mol, respectively, compared to the extended-open (EO) conformer (ΔG^EO^ = 0.0 kcal/mol). For the cyclic RGD peptide (cRGD) as the ligand, the values are ΔG^BC^ = −1.5 kcal/mol, ΔG^EC^ = −1.1 kcal/mol, and ΔG^EO^ = 0.0 kcal/mol corresponding to the population of x(BC):x(OC):x(OE) = 64.3%:31.3%:4.6%. Interestingly, the authors also found that variation in the *N*-glycosylation site number modulates conformational equilibria. The results revealed that bent-closed and extended-closed conformations are stabilized by a lower number of *N*-glycosylation sites on integrin α5β1 [156]. 

The αI domain is the ligand-binding site in the integrins containing this domain. Structural studies of the αI domains (α2, αM, and αL) complexed with a ligand and without a ligand revealed three distinct conformations: closed, intermediate, and open [162,163,164,165]; it was suggested that the closed conformation that lacks a ligand is the most stable [7]. The αI domain possesses a Rossmann fold, and at the C-terminal end of the central β-sheet is a MIDAS binding motif that coordinates a divalent-metal binding site. The crystal structure of αLβ2 also revealed the presence of a ligand-induced allosteric site [166]. In contrast, integrins lacking the αI domain bind ligands in a binding site of the βI domain that is homologous to the αI domain. Readers can find a detailed discussion of the conformational changes of integrins in recent papers [7,13,20,149]. 

### 3.3. Molecular Modeling of Integrins’ Structures

Simultaneously with an effort to describe the 3D structure and conformational dynamic of integrins using experimental methods, molecular modeling methods were applied to provide additional information and aimed to fill the gaps in missing experimental data. The first homology model of an integrin was constructed in 1992 for the α-integrin EF hand-like sequence using the calmodulin sequence as a template [167]. A computational approach was used to design mutations that stabilized the αI domain of the αMβ2 integrin in either the open or closed conformation [168]. The analysis of the predicted mutants revealed that the conformational change in αI domain mediates ligand binding and that computationally proposed ligands are more active than previously suggested ligands.

Up to now, there are no crystal structures reported for the leukocyte integrin α4β1. The first step in generating a complete 3D structure of α4β1 was a homology model of β-subunits, including a bound Mg^2+^ ion [169]. The model was constructed using the I domain of integrin CD11B/CD18 containing Mg^2+^ ion as the template [170]. Then, several steps of restrained energy minimization and molecular dynamics, followed by a final minimization, were used to obtain the final homology model. The ligand-binding mechanism of the α4β1 integrin was studied by docking various molecules, including the vascular cell adhesion molecule (VCAM-1), into the active site of the model. The results shed light on the interactions of β4 with its ligands and explained the binding mechanism of α4β1 with the native ligand VCAM-1. Additionally, a qualitative explanation of the ligand binding selectivity between α4β1 and α4β7 was proposed. 

The solved crystal structures of the complete unconstrained ectodomain plus short C-terminal transmembrane stretches of the &#;V and &#;3 subunits of the αvβ3 integrin [146,153] made it possible to construct a model for the ectodomain of the human αvβ5 integrin [171]. Homology modeling used the crystal coordinates of αvβ3 in its bound conformation as a template. The modeled receptor was refined using energy minimization and molecular dynamics simulations in explicit solvent. The resulting αvβ5 model was used to investigate a ligand binding selectivity toward αvβ3 and αvβ5 by docking various ligands into both integrins. Comparison of both structures and docking results explained the binding differences of both integrins by revealing that ligands with bulky substituents neighboring the carboxylate group are hampered by a “roof” presented on the top of the MIDAS region in αvβ5.

**Figure 6:**
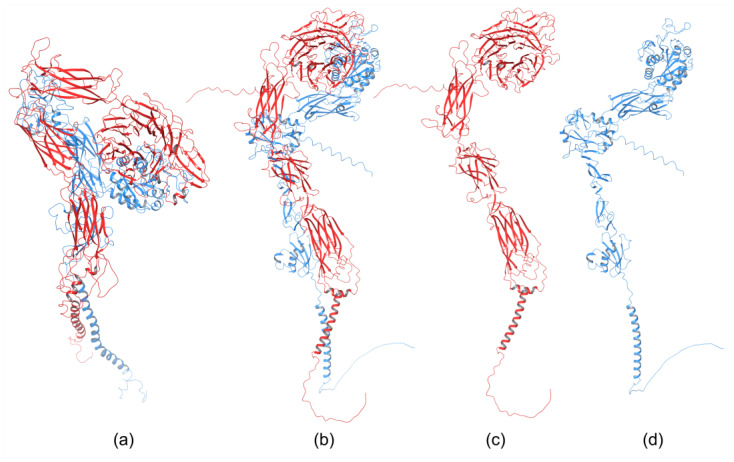
Homology model of (**a**) extended full-length integrin αIIbβ3 [179]; PM0076386 entry in PMDB database, the AlphaFold homology model of (**b**) α4β1, (**c**) the α subunit of α4β1, and (**d**) the β subunit of α4β1 [182].

The homology of the platelet integrin αIIbβ3 has also been reported [172]. At the time of generating the homology model of the αIIb N-terminal portion of integrin αIIbβ3, the high-resolution structures of integrin αIIbβ3 were unavailable. The refined model was validated experimentally. The homology model revealed structural features responsible for the αIIbβ3 integrin function and proposed an interpretation of the role of naturally occurring mutations that produce Glanzmann thrombasthenia. However, more than 38 crystal structures related to integrin αIIbβ3 are now available that provide information on the mechanism of the αIIbβ3 integrin function [20]. The homology model of the extended full-length integrin αIIbβ3 was generated based on the crystal structures of the αvβ3 ectodomain [146,173] and on the β2 PSI/hybrid/I-EGF1-3 construct [174], including of computer models of the TM helices [175]. The model was complemented with *N*- and *O*-glycans, computer models of the TM helices, and NMR structures of the cytoplasmic domains [176,177,178]. The generated models were fit in the EM⁄ET maps, and their hydrodynamic parameters were then computed and compared with the experimental data. Later, the authors [179] refined this model (Figure 6a) using the new crystallographic structure of the integrin αIIbβ3 ectodomain [180] and the NMR structures of its transmembrane/cytoplasmic segments [181]. 

The recently developed deep-learning method AlphaFold [119] has been used to generate a homology model of the α4β1 integrin [182]. AlphaFold produced 25 partially optimized homology structures, including a pLDDT scoring function that evaluates the intra-domain confidence interval. Structures of all homology models were superposed using the USCF ChimeraX program. The analysis of overlapped 3D structures revealed only slight differences in non-structuralized loops. The selected homology model, based on pLDDT, was optimized, and its stability was evaluated with MD simulation using AMBER. The final 3D homology model of the α4β1 integrin is shown in Figure 6b, together with the homology structures of the α subunit (Figure 6c), and β subunit (Figure 6d).

## 4. The Biological Function of Integrins

Integrins possess a rare ability to transduce signals across the plasma membrane in both directions. The so-called outside-in signaling is mediated by ligand binding to an integrin ectodomain, upon which a conformational change occurs and a signal is transmitted to the cell. Conversely, interactions of cytoplasmic domains with cytoskeleton proteins or signaling molecules dynamically regulate the activation or deactivation of integrins by so-called inside-out signaling [7,183]. Integrins interact with a vast number of proteins from the extracellular matrix, with molecules on the surface of other cells and soluble proteins, and thus mediate a wide range of physiological processes. After their activation, integrins form adhesion complexes, the so-called adhesome that transduces adhesion-dependent signals to control many cellular functions [184]. Integrins transduce signals bi-directionally through the plasma membrane between extracellular and cytoskeletal space [18,185,186]. Extracellular ligand binding to the integrin headpiece (i.e., fibronectin or collagen) or an external force [12] triggering signal transmission from the extracellular to the cytoskeleton is called outside-in activation, while the binding of intracellular activators (i.e., talin or kindling) to the cytoplasmic tails leading to signal transmission from inside the cell to outside the cell is called inside-out activation. The integrin signaling is associated with conformational changes in both subunits of integrins and integrin clustering, and is responsible for activating integrins. Various pathways were discussed in the literature [11,187], and molecular dynamics simulation methods have been used to decipher the conformational dynamics of integrins during activation. The MD simulations also provided valuable information on the atomic level and complemented experimental data about the dynamics of integrin–ligand interactions.

### 4.1. Molecular Simulations of Integrins’ Conformational Dynamics

Interestingly, probably the first dynamics study on integrins was the use of a Brownian dynamics algorithm to simulate the cytoskeleton-mediated transport of an integrin on the dorsal surfaces of migrating fibroblasts published in 1994 [188]. The results suggested that besides a diffusion/limited process, direct transport is also necessary for the delivery of integrins to the adhesion area. 

The integrins αvβ3 [131,187,189,190,191,192,193,194,195,196] and αIIβ3 [185,197,198,199,200,201,202] are the two most investigated integrins by molecular dynamics. This is quite understandable, as these integrins belong to the RGD group with several crystal structures available and are associated with various human diseases. The αvβ3 and αIIβ3 integrins both lack the αI domain. The prevailing MD simulations have focused on understanding activation and transition from bent to extended conformations initiated by inside-out and outside-in signaling.

The βI domain in integrins lacking the αI domain contains three metal binding sites. The presence of a MIDAS metal ion was confirmed by the crystal structure of the αIIβ3 and αvβ3 integrins [173,203]. Two additional binding sites close to MIDAS were designated as AMIDAS and ligand-associated metal binding sites (LIMBS). To clarify the function of LIMBS on the binding behavior of physiological ligands to β3 integrins, the MD and steered MD (SMD) simulations were combined with the experiment [204]. The starting structure for simulations was the crystal complex of the αIIβ3 integrin fragment with eptifibatide [203] and its β3 LIMBS D217A mutant. The experimentally obtained data suggested that the D217A mutation affected β3 structure and the binding of ligands. On the other hand, SMD simulations demonstrated that removing the metal ion from LIMBS decreases the ligand binding affinity. Moreover, the more significant effect was seen without metals in the MIDAS and LIMBS, suggesting that the LIMBS D217A mutant lacked both metal ions. The FMO PIEDA analysis demonstrated that the MIDAS and LIMBS ions are more critical for binding eptifibatide than is the ADMIDAS ion [205].

The interactions between the cyclic Arg-Gly-Asp (RGD) peptide and divalent cation within the integrin binding site were explored using equilibrium MD simulations [190]. In addition, non-equilibrium SMD simulations were used to describe how the αvβ3-RGD ligand complex dissociates under force. Structural models for these simulations were based on the crystal structure of the αvβ3 integrin in complex with the RGD ligand [173]. Computations revealed that the key interaction between the αvβ3 and RGD ligand is between the metal and Asp(RGD) and demonstrate a crucial role of a single water molecule stabilizing the αvβ3-RGD ligand complex by simultaneously binding to a MIDAS divalent metal ion and Asp(RGD). It is noteworthy that simulations also found that Asp(RGD) interacts with both metal ions from MIDAS and LIMBS sites.

The role of binding a fibronectin (Fn) module to the headpiece of the αvβ3 integrin on integrin activation was investigated by performing MD and SMD simulations [195,206]. For the simulations, the RGD ligand in the crystal structure [173] was replaced by the RGD-containing 10^th^ type III fibronectin module (FnIII_10_). Simulations showed that the closed βI/hybrid domain opens spontaneously and is accelerated by binding FnIII_10_ and ligand-mediated forces.

Molecular insight into the outside-in activation of the αvβ3 integrin triggered by binding glycoprotein fibronectin models 9 and 10 (FnIII_9_ and FnIII_10_) was obtained using MD simulations [207,208]. The results showed that the binding of FnIII_9_ and FnIII10 induced by the presence of Mn^2+^ is associated with a change in the conformational dynamics observed in both αv and β3 domains. These changes contributed to αvβ3 integrin activation, resulting in closed to extended conformation change. In this conformational change, the movement of the α1 helix in the βI domain played a relevant role. MD simulations were performed on the full-length ectodomains, but the parts of transmembranes and cytoplasmic domains were not considered.

In the integrin lacking the αI domain, the βI domain flanked by hybrid domains contains the primary binding site of the integrin where the ligand binds via MIDAS. MD simulations were used to characterize the movement of βI/hybrid domains of the β3 subunit of the αvβ3 integrin in both open and closed headpiece conformations [193]. Based on MD simulations, the authors proposed that α7 and α1 helices from the βI domain initiate in a simultaneous action a significant interdomain conformational transition observed in integrin activation.

**Figure 7:**
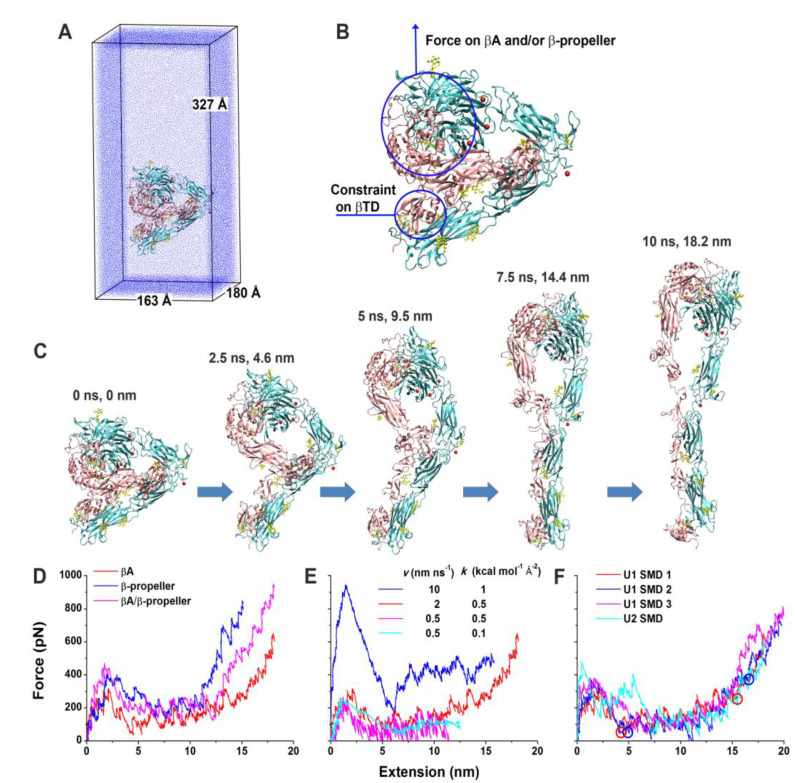
SMD simulations of the integrin αvβ3 unbending under an external force. (**A**) U1 in the enlarged water box for unbending simulations. (**B**) Illustration of force application on the head and constraint on the βTD in the SMD simulations of U1 and U2. (**C**) Snapshots of a representative unbending process (U1 SMD 1) taken at indicated times and extensions. (**D**) Force-extension curves in the constant-velocity SMD simulations of U1 by pulling the βI and β propeller domains with a 2 nm ns^−1^ pulling speed and a 0.5 kcal mol^−1^ Å^−2^ spring constant. (**E**) Force-extension curves in the constant-velocity SMD simulations of U1 by pulling the βI domain with indicated pulling speeds and spring constants. (**F**) Force-extension curves for three constant-velocity SMD simulations of U1 and one constant-velocity SMD simulation of U2 with a 2 nm ns^−1^ pulling speed and a 0.5 kcal mol^−1^ Å^−2^ spring constant. Red and blue circles indicate respective structures along the unbending pathways from the trajectories of the U1 SMD 1 and 2 that were selected as starting structures for free MD simulations. The left two represent partially-extended structures, and the right two represent fully-extended structures. The red curves in panels D–F are all for the U1 SMD 1. Reprinted with permission from ref. [194].

Forced unbending of a complete ectodomain of the αvβ3 integrin in both unliganded and liganded forms was studied using an all-atom explicit solvent MD [194]. In the closed conformation of the αvβ3 integrin, βI and hybrid domains were based on the crystal structure of the αvβ3 integrin ectodomain [209]. The open conformation was generated from the open headpiece of the αIIbβ3 integrin [203]. Simulations of pulling the head of both unliganded and liganded forms of the αvβ3 integrin using a force induced a gradual transition from the bent to the extended conformation. At the same time, the domains were not significantly distorted. The results of the simulation of the unbending transition for the unliganded form is shown in Figure 7. The simulation showed the significance of the nonpolar interaction between the hybrid and EGF4 domains for stabilizing the bent conformation and that in the extended conformation, Asp457 from the thigh domain moved to coordinate the Ca^2+^ ion at the αv subunit, suggesting that these polar interactions stabilize the extended conformation. 

The self-association dynamics of the αIIβ3 and αLβ2 integrins transmembrane domains have been investigated using coarse-grain (CG) MD simulations [197]. High sequence homology between both integrins allowed for the generation of the αLβ2 homology model based on the crystal structure of αIIβ3 [181]. Compared to αIIβ3, the αLβ2 integrin contains in the TM sequence a polar residue in its αL (Ser) and β2 (Thr) subunit, respectively, that is involved in hydrogen bonding. The TM models were embedded into the DPPC lipid bilayer and a periodic cubic water box. CG MD simulations using the GROMACS package [210] and Martini force field [211] revealed that polar interactions play an important role in packing helices. Simulations showed that the αLβ2 TM packing is almost optimal and is more specific, while the packing of the αIIβ3 was found to be suboptimal. Simulations of the T686G mutants that have a disrupted hydrogen bond showed a poorer subunits association supporting a significant role of polar residue on the association of TMs. The calculated free energy of the association predicted a lower minimum for the αLβ2.

MD simulation and homology modeling were carried out on the complete extracellular domain of the β3 subunit of the αIIβ3 integrin [198,212]. The results indicated that the mutant at the 33 position does not affect the conformational dynamics of β3. The main effect was a change of conformational equilibrium to more rigid structures, which might influence the binding properties of the αIIβ3 integrin in a studied mutant.

All-atom MD simulations investigated the inside-out activation of the platelet integrin αIIβ3 triggered by talin [200]. MD simulations were performed on the constructed model of the entire TM, cytoplasmic tails (CT) of the αIIβ3 integrin embedded in a lipid membrane, the explicit water environment, and also in the presence of the talin-1 F2 and F3 subdomains using the CHARMM27 force field [213]. The 5μs simulations provided insight into the inside-out activation at the atomic level, suggesting a preferred conformation of the entire TM-CT αIIβ3 domain and proposed crucial interaction in the αIIβ3-talin complex. 

Structural changes in the transition from low-affinity bent conformation to high-affinity extended conformation were studied on the full-length αvβ3 integrin using all-atom MD and a coarse-grained heterogeneous elastic network model (hENM) [189]. The full-length αvβ3 integrin was constructed from crystal structures of the bent headpiece [153] and transmembrane and cytoplasmic parts [214]. Simulations provide new information about structures along switchblade and deadbolt pathways from bent to extended conformation. The results also support the hypothesis that weakening long-range interactions between distant domains that binding activators can trigger are responsible for transitioning from bent to extended conformation.

Integrins mediate extracellular matrix stiffness sensing by cells and serve as sensors of mechanical signals [12]. It was suggested that the integrin provides a rigidity-sensing mechanism through conformational dynamics during ligand binding [215]. Coarse-grained MD simulations [191] were conducted to investigate how forces applied to the αvβ3 integrin influence its conformational dynamics and mechanical signaling (mechanotransduction). The CG computational model of the wild-type αvβ3 integrin and several mutants were developed from crystal [214] and NMR [214] structures. The results of CG MD simulations were backmapped to all-atom models, inserted into a lipid bilayer, and hydrated. Then, constant-force SMD was performed on systems containing 1.9 million atoms for the wild-type αvβ3 integrin and 2.2 million atoms for the mutant. The computations revealed that the activated mutant requires lower force for transition to a high-affinity conformation than does the wild-type integrin. Consequently, cellular-stiffness-sensing correlates with integrin conformational flexibility, supporting the role of integrins as true mechanosensors [191].

The structure of the entire model of the αvβ3 integrin was generated [187] using crystal structures of the αvβ3 integrin ectodomain [216], and the homology model of the transmembrane domain based on the crystal structure of the αIIbβ3 integrin [181]. Then, the αvβ3 integrin model was glycosylated and imbedded into a dipalmitoyl-phosphatidylcholine membrane and used for MD and SMD simulations carried out with the software package GROMACS [210]. Outside-in activation was studied using an applied force to the extracellular domain, and the inside-out activation was investigated by binding talin to the connected or separated cytoplastic tails. Simulations supported the switchblade model, similar to that suggested for the αIIbβ3 integrin [186], for both the outside-in and inside-out activation.

A mechanism of the inside-out signaling of integrins mediated by the interaction of Kindlin2 to the cytoplasmic domain was investigated using “rampclamp” SMD simulation [217]. The crystal structure of the Kindlin2 complex with the β3 integrin [218] imbedded in a rectangular box of water was utilized as the starting structure. The NAMD software package [102,103] and CHARMM27 force field [213] were used for the calculations. Simulations showed that 17 hydrogen bonds (five strong) altogether were responsible for the stability of the equilibrated complex and integrin activation. SMD simulations using various forces revealed the catch-slip bond mechanism for the Kindlin2-β3 integrin interaction.

Binding of the RGD containing the 10th domain of fibronectin (FnIII10) to extended conformations of the αvβ3 integrin was investigated using MD simulations. A crystal structure of the FnII10-αVβ3 integrin complex [219] was used as a starting structure of the open headpiece conformation. The structure of the extended conformation was constructed using βI and hybrid domains from the crystal structure of the open form of αIIβ3 [181]. Simulations showed that efficient fibronectin binding requires open conformation of the αVβ3 integrin.

A combination of AFM measurement and MD and SMD simulations [220] was used to elucidate the strength of binding a β2 subunit of the αLβ2 and αMβ2 integrins to intercellular adhesion molecule-1 (ICAM-1). The binding strengths of complexes of β2 subunits with the Ca^2+^ ion in the MIDAS were evaluated using SMD simulations. Simulations models were generated from crystal structures of the αLβ2 and αMβ2 integrins with ICAM-1 [220,221]. Simulations showed that interactions in the αMβ2-ICAM-1 complex are stronger than those in the αLβ2-ICAM-1 complex, in agreement with AFM data. These results suggest that neutrophil adhesion under shear flow in the blood is dominated by ICAM-1 interactions with the αLβ2 integrin.

### 4.2. Integrins’ Activation

**Figure 8:**
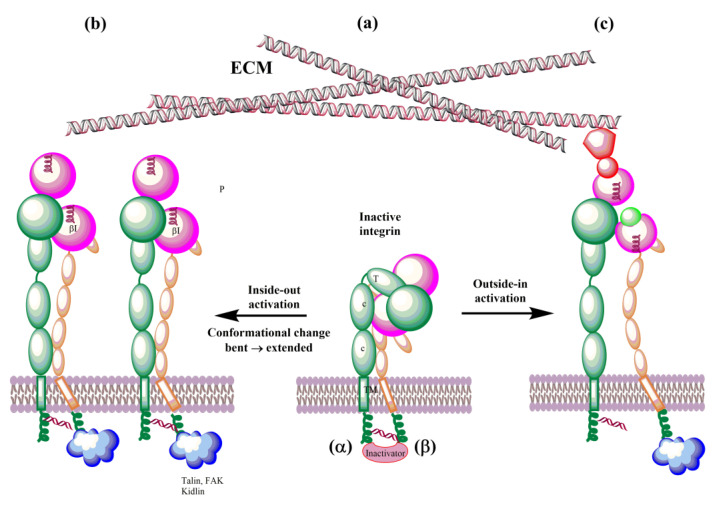
Schematic representation of bidirectional integrin activation. (**a**) Inactive integrin; (**b**) inside-out activation triggered by binding ligands to the cytoplasmic tail of β subunit; and (**c**) outside-in activation by interactions with ECM.

In the last decade, understanding of the details of both biochemical [13,157,222,223,224,225] and mechanical [12,226,227,228] integrin signaling (Figure 8) has significantly advanced. Integrins exist in an equilibrium of several conformations that represent different affinity states. The above-selected MD papers helped to understand their conformational dynamics and regulation. Simulations of integrins’ interactions with talin, kidlin, and fibronectin [193,195,200,206,207,208,217,219] and simulations of a force influence on the conformation of integrins [187,190,191,194] decipher interactions associated with the outside-out, inside-out, and mechanical signaling (mechanotransduction) at the atomic level. 

The biosynthesis of integrins is concluded by post-translational modification, such as glycosylation, in the endoplasmic reticulum and Golgi apparatus. The mature integrins are then transported to the plasma membrane in the bent inactive conformation (Figure 8a). On the membrane, integrins must be activated to be involved in interactions with ECM proteins. Activation can be simplified as the transit from the inactive bent-closed conformation through the extended-closed and extended-primed to the high-affinity extended-open [11,12,225]. The first step of the activation in the inside-out mechanism involves the binding of talin to the cytoplasmic tail of the β subunit. This binding with the extracellular metal and ECM forces an unbent integrin, replaces the intracellular inhibitor, and separates α and β subunits. Inside-out signaling is supported by the binding of focal adhesion kinase (FAK) and kidlin and, thus, regulates affinity for extracellular ligands and clustering of integrins (Figure 8b). In addition, other effectors, such as paxillin, actin, and myosin, influence the adhesion maturation of integrins. Interactions of integrins with adhesion proteins control various signaling pathways, called outside-in signaling, crucial for multiple cell processes dependent on integrins. In outside-in activation, the binding of ligands triggers a conformational change of the βI domain, referred to as headpiece opening.

On the cell surface, integrins are under the influence of the force induced by glycocalyx covering the cell surface and the forces between cells and ECM. These forces influence their conformation equilibrium and, thus, ligand binding properties, activation, and clustering. Therefore, an external force operating from ECM regulates integrin functions and is called the outside-in activation (Figure 8c). MD simulations support the role of force [180,187,191,194,206]. Structural changes observed in the transition of integrins from the low-affinity bent conformation to the high-affinity extended conformation during their activation are relevant in designing modulators of their biological function with potential therapeutic use. The αvβ3 and αIIβ3 integrins dominate MD simulations due to a number of their solved 3D structures. However, with an increasing number of X-ray or NMR 3D structures and reliable homology models, it is reasonable to assume that the remaining integrins will also be studied using molecular modeling methods.

## 5. Integrins as Therapeutic Targets

### Integrins in Diseases

Integrins, as transmembrane glycoproteins located on the surfaces of the cells, recognize many physiological ligands [229]. They bind through their ectodomains with numerous ligands and, thus, are involved in cell–cell and cell–ECM interactions influencing cell migration and ECM assembly and remodeling. Among the most relevant ligands belong ICAM-1 (Intercellular Adhesion Molecule 1; also known as CD54), VCAM-1 (Vascular Cell Adhesion Molecule 1; CD106), MAdCAM-1 (Mucosal Addressin Cell Adhesion Molecule 1), E-cadherin, PECAM-1 (Platelet Endothelial Cell Adhesion Molecule 1; CD31), EPCR (Endothelial Cell Protein C Receptor), thrombomodulin, fibronectin, collagen, and irisin [230]. The cytoplasmic domain of integrins also interacts with many cytoskeletal proteins and signaling molecules. These interactions mediate fundamental cell processes associated with diverse physiological and pathological pathways. Though integrin–ligand interactions play a pivotal role in maintaining the health conditions of various tissues, their aberrant activation is detrimental in multiple diseases, including development, immunity, hemostasis and thrombosis, inflammation, angiogenesis, tumor growth and metastasis, multiple sclerosis, inflammatory bowel disease, nephritis, osteoporosis, sickle cell disease, and fibrosis [9,13,24,231,232]. Many papers exist regarding the role of aberrant integrin adhesion and signaling in the pathogenesis of many human diseases. It is beyond this paper’s scope to discuss this complex area of research in detail. Therefore, the following sections only briefly discuss the importance of integrins in various diseases, and readers may find more detailed insight in available reviews.

**Figure 9:**
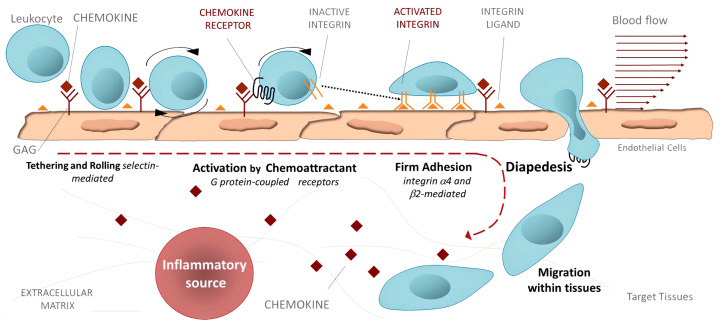
Leukocyte adhesion cascade: the multi-step recruitment process from blood to target tissues in the inflammatory source. Reprinted with permission from ref. [234].

*Inflammation.* Activated integrins are involved in leukocyte extravasation from blood to inflamed tissues. This process consists of multiple sequential molecular interactions called leukocyte adhesion cascade [150,233]. Circulating leukocytes interact during tethering and rolling with selectins on the activated endothelium. These contacts are identified by chemokines, which trigger inside-out activation (by binding effectors to the cytoplasmic tail of the β subunit) of leukocyte integrins (e.g., αLβ2, αMβ2, α4β1, and α4β7) that then bind to their counter-receptors on the endothelium, including ICAMs and VCAMs. Binding these adhesion ligands stabilizes the high-affinity integrin conformation and strengthens the binding of leukocytes to the endothelium. Firmly bound leukocytes crawl along the endothelium and finally migrate through the endothelium to inflamed sites [183] (Figure 9).

Integrins are crucial components of the leukocyte adhesion cascade responsible for proper leukocyte homing in inflammatory responses. Their role is documented by patients with leukocyte adhesion deficiency (LAD) syndromes who suffer from recurrent infections and bleeding disorders. It was discovered that a mutation in β2 integrins is responsible for LAD affecting the interaction with kindlins-3, and as a result, leukocytes cannot get to the inflammation site [235]. A complete failure of platelet aggregation to form a clot caused by mutations of the αIIbβ3 integrin is characteristic of Glanzmann’s thrombasthenia [236]. Abnormal bleeding that can be life-threatening is a typical symptom of patients suffering from Glanzmann’s thrombasthenia. Another genetic disease is Epidermolysis bullosa, a connective tissue disorder that causes your skin to blister and tear easily, caused by a mutation of the α6β4 integrin [237]. Symptoms are often severe with life-threatening complications. Integrins are crucial in preventing chronic inflammation by removing apoptotic neutrophils by macrophages in acute inflammation, an efferocytosis process [238]. 

*Inflammatory bowel diseases*. Leukocyte integrins play a prominent role in inflammatory bowel diseases (IBDs), including Crohn’s disease (CD) and ulcerative colitis (UC). Uncontrolled inflammation of the gastrointestinal tract is typical for IBDs [239,240]. The migration of activated T-lymphocytes to the intestinal vasculature is mediated by interactions of α4β1, α4β7, and αEβ7 integrins with their ligands VCAM-1, MAdCAM-1, and E-cadherin. In the inflamed gut of IBDs patients, an increased number of VCAM-1 and MAdCAM-1 ligands were observed that contributed to the increase of pro-inflammatory lymphocytes, which are retained through enhanced interactions between the αEβ7 integrin and E-cadherin. Thus, aberrant interactions of α4β1, α4β7, and αEβ7 integrins with their ligands VCAM-1, MAdCAM-1, and E-cadherin play critical roles in the pathogenesis of BDIs [239]. Therefore, the therapy based on inhibiting these interactions may be beneficial for treating patients suffering from BDIs.

*Arthritis*. Inflammation of the synovium tissue is characteristic of chronic inflammatory arthritides. Rheumatoid arthritis (RA) is the best-studied disease in this group [241,242,243]. In RA, enhanced pro-inflammatory cell levels cause overexpression integrin receptors and their ligands [244]. The analysis of integrin distribution in synovial tissue of RA revealed [243] an increased expression of collagen-, laminin-, and fibronectin-binding integrins, especially those containing α5, αv, and β1 subunits. Additionally, an upregulation of the αLβ2 (LFA-1) integrin that enhances the migration of immune cells into the synovial tissue was observed [243]. Enhanced levels of these integrins causes the overproduction of matrix-degrading enzymes and fibroblasts that degrade cartilage, and thus preserve RA. All these findings suggest integrins’ crucial role in RA disease that can be restrained with integrin inhibitors.

*Fibrosis*. Five integrins containing the αv subunit (αvβ1, αvβ3, αvβ5, αvβ6, and αvβ8) have been identified to play a relevant role in fibrotic diseases [245] in several organs, including the heart, blood vessels, lung, kidney, liver, and skin [246]. Typical for fibrosis is ECM stiffening with loss of elasticity and excessive tissue deposition with a debilitating condition [247]. Under chronic injury or inflammation, integrins activate pro-fibrotic transforming growth factor β (TGFβ). Induced fibroblasts upregulate ECM production, leading to fibrosis progression. It was found that αv integrins are upregulated in fibrotic diseases, and studies using knockout mice demonstrated that deletion of αv integrins might attenuate fibrosis progression [248].

*Atherosclerosis*. Integrin signaling plays a crucial role in atherosclerosis, a chronic inflammatory disease affecting large arteries [249,250]. The binding of the αIIbβ3 integrin with fibrinogen is involved in platelet aggregation, and β2 integrins (αM4β2 and αLβ2) control macrophage binding. An overexpression of integrins and their ligands was observed in atherosclerosis [249]. For example, an upregulation of the α4β7 integrin and its ligands VCAM-1 and MAdCAM-1 was found in atherosclerosis, and the atherosclerotic plaque area was significantly reduced in the α4β7 deficient mice [251,252]. In addition, attenuated atherosclerosis was observed upon deletion of other integrins, such as the leukocyte αXβ2 [253], αvβ3 [254], and α5β1 [255]. Therefore, the inhibition of these integrins has the potential to reduce the progression of atherosclerosis.

*Eye diseases.* Integrins play an essential role in normal development and the development of pathological processes in the eye [26]. For example, the αvβ6 integrin is a key player in corneal fibrosis [256]; integrins α1, α3, α4, αL, β1, β3, and β4 were upregulated in the heredity eye disease Fuchs’ corneal dystrophy [257]. The αL integrin plays a vital role in dry eye diseases, and its inhibition significantly improves ailments [258]. In glaucoma, the αvβ3 integrin was upregulated in retinal ganglion cells and the glial cells of the nerve head after nerve crush in mice [259]. The examples mentioned above documented some eye diseases associated with the deregulation of integrins.

*Cancer*. A multistep process of cancer development includes tumor initiation and sustainable chronic proliferation, local invasion and intravasation into blood, surviving circulation, adhesion to the endothelium, extravasation, initial seeding, and proliferation in the target tissue [260,261,262,263]. Many studies have indicated that integrins mediate various aspects of these steps [23,264,265,266,267,268,269], and below, we present only a few selected examples. Biochemical and genetic studies have documented aberrant integrin activity in cancer cells associated with an altered expression of integrins, which is dependent on the cancer type and the stage of the disease [23]. A high abundance of various β1, β4, and αv integrins (α3β1, α4β1, α5β1, α6β4, αvβ3, αvβ5, αvβ6, and αvβ8) is associated with metastasis and frequently correlates with poor prognoses [23,267]. However, the role of integrins is not straightforward. For example, although β1 integrins play a crucial role in cancer development and the α3β1 integrin is vital for mammary cancer [270], the α2β1 integrin is a metastatic suppressor in breast cancer [271].

Genetic studies have revealed that the β4 integrin is necessary for tumor initiation and progression in mammary and skin tumorigenesis [265]. Additionally, it was found [269] that an overexpression of the αvβ3 integrin plays a vital role in developing tumor-initiating cells in lung and pancreatic cancers. These cells are assumed to contribute to cancer relapse after the initial response to treatment. Furthermore, the αvβ3 integrin was found to mediate the resistance of tumor-initiating cells to tyrosine kinase inhibitors through the activation of NF-κB in a ligand-independent manner [272].

Cancer metastasis is a complex multi-step process, and from a vast number of primary tumors, only a tiny number of metastases develop. To form metastasis in nearby or distant organs, cancer cells have to accomplish all of several consecutive steps: detachment from the primary tumor, intravasation to the blood vessel, survival of circulation in blood and adhesion to the endothelium, extravasation from the blood into the target organ, and proliferation in the organ microenvironment [260,263]. Accumulating experimental evidence showed that during the circulation in the blood, cancer cells utilize a similar mechanism used by leukocytes in the inflammatory cascade [273,274]. Various adhesion molecules mediate the transendothelial migration of cancer cells, including activated integrins of cancer cells, such as α4β1 binding to endothelium ligand VCAM-1 and αLβ1 binding to LCAM-1.

However, the role of integrins is more complex, and some data suggest that laminin-binding integrins α3β1 and α6β4 might have an inhibitory effect on cancer metastasis [275]. The dual role of the α3β1 integrin was shown in breast cancer. The absence of integrin α3β1 reduced the survival of mice, and increased tumor growth was observed [276]. Similarly, the α3 subunit of the α3β1 integrin interacts with various ECM ligands, and its function depends on the cancer type. In patients with hepatocellular carcinoma (HCC), the expression of α3 negatively correlated with tumor growth and metastasis [277]. An opposite functioning of α9 was observed in breast cancer, where knocking out α9 significantly reduced tumor growth, angiogenesis, and metastasis [278].

Integrins are also involved in ECM remodeling to induce cancer cell invasion, with cancer-associated fibroblasts (CAFs) playing a vital role. It was found [279] that the αvβ3 integrin expressed by CAFs participates in CAFs’ assembling of fibronectin and metastasis. In addition, other integrins, such as the α5β1 integrin [280], αvβ6 integrin [281,282], and α9β1 integrin, promote the recruitment of CAFs. Angiogenesis supplies nutrition for tumor survival and supports tumor cell transfer into blood vessels for circulation. Three endothelial integrins, namely αvβ3, αvβ5, and α5β1 mediate tumor angiogenesis [283]. It has been shown that tumors use integrin-ECM interactions as one of the strategies to escape anti-tumor therapies [284]. To achieve this goal, tumors overexpress integrins, such as β1, and activate signaling pathways that block the effect of drugs [285,286].

*Integrins as a route to invasion by viruses and bacteria.* Various pathogens can exploit integrins as receptors to attach and enter the host cells; for review, see references [287,288,289,290]. Over time, viruses have evolved multiple mechanisms to colonize host cells. The binding to the host is the first step of virus entry (internalization), and among different receptors, viruses utilize integrins.

Several viruses display on the viral surface proteins containing amino acid moiety RGD, which they use for binding with RGD-binding integrins [289] (αvβ1, αvβ3, αvβ5, αvβ6, αvβ8, α8β1, and αIIbβ3). Among those, many adenoviruses interact with αv integrins as documented by the solved structure of the complex with the αvβ5 integrin by cryoelectron microscopy [291]. The binding starts virus internalization, and it was shown that inhibition of binding resulted in a significant decrease in viral infection [292]. Interestingly, adenovirus binding also induces the clustering of integrins that enhance infection. Similarly, several members of the Herpesviridae family, such as Kaposi’s sarcoma-associated herpes virus or human herpes virus 8, utilize the αvβ3 integrin [293]. The integrins αIIbβ3 and αvβ3 function as receptors for pathogenic strains of hantaviruses, while non-pathogenic strains of the Prospect Hill virus utilize the β1 integrin [294,295]. Coxsackievirus, a member of the enterovirus family, uses the αvβ6 integrin for cell entry [296]. Interactions of retrovirus human immunodeficiency virus 1 (HIV-1) with the α4β7, αvβ5, αvβ3, and α5β1 integrins are critical for cell entry [297,298,299]. Among other RGD-binding viruses, deadly Ebola virus interactions with the α5β1 integrin are essential for fibroblast infection [300]. Other viruses using RGD moiety for engagements with host cells include Zika virus [301] (αvβ5), rotavirus [302] (αvβ3), and foot-and-mouth disease [303] (αvβ6). Recently, it was suggested that SARS-CoV-2 might also use RGD-binding integrins as cell receptors through interactions with spike protein [304,305].

Not all viruses recognize RGD moiety for their interactions with integrins. An alphavirus Ross River virus associated with polyarthritis utilizes the binding of integrins α1β1 and α2β1 for cell entry and infection [306]. The role of the α2β1 integrin is supported by blocking infection with function-antibodies against α2β1 [306]. Rotavirus spike protein uses different spike amino acids moieties to enter a cell: the YFL domain binds with the α4β1 and α4β7 integrins [307], and the GPR moiety interacts with the αXβ2 integrin [308,309]. Human echovirus, which is associated with meningoencephalitis, utilizes for successfully infecting cell clusters of the α2β1 integrin [310]. Interestingly, HIV-1, in addition to binding RGD-binding integrins, also uses interactions with the α4β7 integrin for efficient cell-to-cell spreading [298].

Integrin receptors are also vital for many bacterial infections, and in the following, only some examples will be presented. More details can be found in reviews [288,290]. Some bacteria use an adhesion, a protein expressed on their surface, to interact with integrins on host cells to initiate cell entry. For example, *Yersinia* bacteria cause pain and tenderness in the abdomen, nausea, and diarrhea, and use the protein invasin to interact with five β1 integrins, namely α3β1, α4β1, α5β1, α6β1, and αvβ1, for efficient host cell entry [311]. *Helicobacter pylori* is linked to various stomach diseases, and the host clustered β1 integrins attach bacteria through a type 4 secretion system to the cell membrane [312]. *Borrelia burgdorferi* bacteria is a source of Lyme disease, and a membrane protein P66 binding to β3 integrins has been identified as the mechanism of bacteria adhesion [313]. 

Some bacteria express proteins that bind to the protein fibronectin from ECM and through fibronectin to host cell integrins in the so-called sandwich model [314]. For example, *Staphylococcus* bacteria cause mucosal or septicemic infection and express two fibronectin-binding proteins, FnbpA and B. These proteins interact with the α5β1 integrin and can be inhibited by RGD peptides [315]. Streptococcus bacteria use a similar mechanism responsible for acute pharyngitis in humans [316] and by *Porhyronomas* bacteria that causes periodontitis [317]. A common bacterium, *Pseudomonas aeruginosa,* causes acute or chronic lung infections and employs interactions with α5β1 and αvβ5 integrins and their ligands fibronectin and vitronectin to invade host cells [318]. *Neisseria* bacteria cause sexually transmitted gonorrhea disease (*N. gonorrhoeae*) and meningitis (*N. meningitidis*). Infections by *Neisseria* commence with an attachment to host cell surfaces. Then, the host cell receptors trigger signaling, activating the α5β1 and αvβ3 integrins with the following cell entry [319]. The above-discussed example illustrates the crucial importance of integrins’ recognition in viral and bacterial infections. In many cases, they mediate attachment, internalization, and tissue. Thus, they represent potential targets for therapeutic intervention.

## 6. Integrin-Based Therapeutics

The above examples illustrate integrins’ association with various physiological and pathologic processes and diseases. Therefore, it is unsurprising that integrin-related diseases are an attractive target for drug development [14,24,27,231,320,321,322]. Different therapeutics have been designed to intervene in integrin functions by restraining or stimulating cell penetration into the tissues, including antibodies, small non-peptide molecules, and peptides [24]. Not all academic and industrial research efforts to develop integrin-based therapeutics have been successful. In the last 30 years, there have been many agents in clinical trials, but only seven approved integrin-based drugs [24].

### 6.1. Marketed Drugs

**Figure 10:**
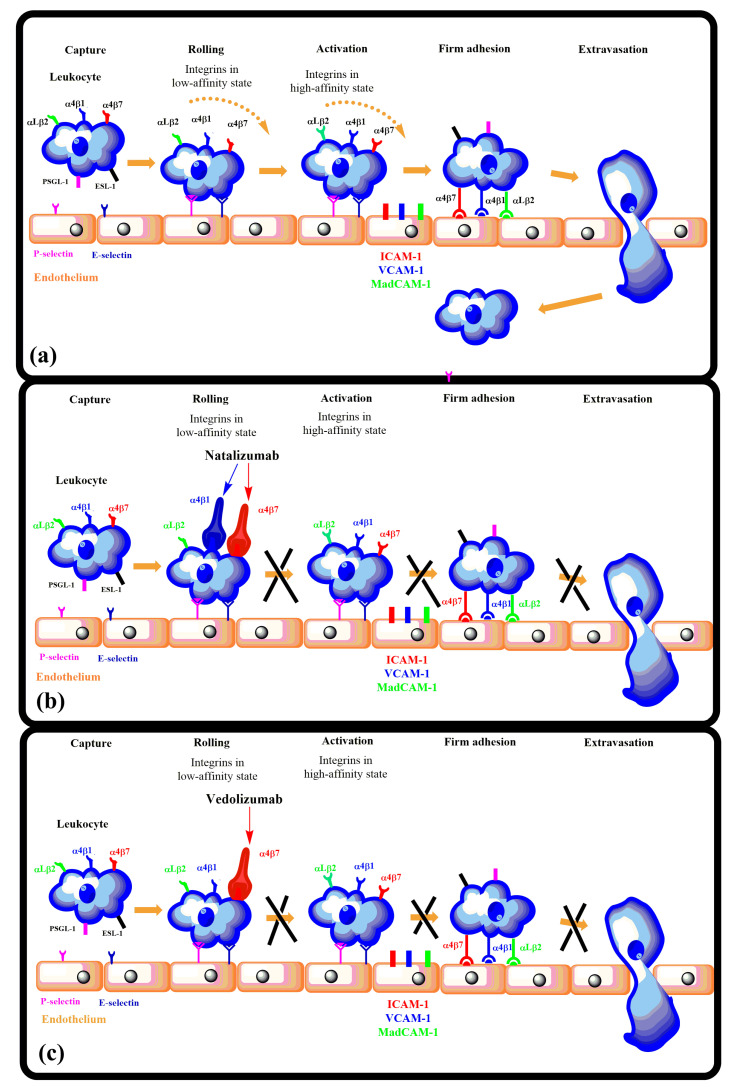
The therapeutic action of natalizumab and vedolizumab. Integrin antagonists inhibit leukocyte migration into endothelium: (**a**) leukocyte adhesion cascade, (**b**) natalizumab binds to both the α4β1 and α4β7 integrins, thus blocking leukocyte adhesion, (**c**) vedolizumab binds only to the α4β7 integrin, which minimizes potential side effects. Based on ref. [324].

The first drug developed and approved in 1994 was the antibody abciximab (ReoPro), a platelet aggregation inhibitor that is a pan-β3 antagonist used to inhibit the binding of the αΙΙbβ3 integrin to fibronectin. This interaction prevents platelet aggregation, causing blood clots within the coronary artery and targeting acute coronary syndrome and thrombotic cardiovascular syndrome. Another three drugs, efalizumab, natalizumab, and vedolizumab, are also antibodies. Efalizumab (Raptiva) is a humanized monoclonal antibody (mAb) designed to treat the autoimmune disease psoriasis. Efalizumab blocks extravasation of the lymphocyte by inhibiting the αLβ2 integrin. Efalizumab was associated with fatal brain infections and was withdrawn from the market in 2009 [323]. Natalizumab (Tysabri) is a humanized mAb used to treat multiple sclerosis (MS) and Crohn’s disease. Natalizumab reduces the homing of T cells to the gut by inhibiting ligand binding to α4β7 and α4β1 integrins. Natalizumab is clinically effective but is associated with severe adverse effects, including fatal neurological disease and progressive multifocal leukoencephalopathy. Interestingly, natalizumab was withdrawn four months after its approval in 2005, but in 2006 returned to the market for MS. Vedolizumab (Entyvio) is a humanized mAb targeting the α4β7 integrin. It treats inflammatory bowel disease (IBD), including ulcerative colitis and Crohn’s disease. Figure 10 illustrates the mechanism of the therapeutic action of natalizumab and vedolizumab.

**Figure 11:**
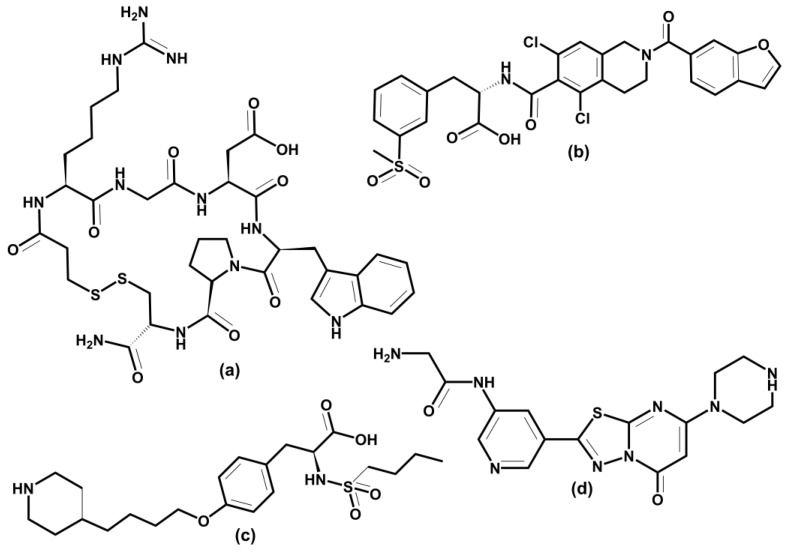
Schematic representation of the three small molecule marketed drugs: (**a**) eptifibatide, (**b**) tirofiban, (**c**) lifitegrast, and (**d**) zalunfiban (RUC-4), compound in the Phase 3 clinical trial.

Eptifibatide (Integrilin), tirofiban (Aggrastat), and lifitegrast (Xiidra) are three small molecule integrin antagonists that entered the market (Figure 11). Eptifibatide is a heptapeptide RGD mimetic and binds to platelets through the αΙΙbβ3 integrin; it was approved in 1998. Similarly to abciximab, eptifibatide prevents causing thrombus in a coronary artery. It is used to reduce the risk of acute cardiac ischemic events. The second small molecule drug, tirofiban, employs the same mechanism and has the same indication as abciximab and eptifibatide. It was approved in 1998. The third small molecule drug, lifitegrast, was approved in 2016 and is an inhibitor of the αLβ2 integrin. Lifitegrast blocks the binding of T lymphocyte’s integrin αLβ2 to its ligand ICAM-1 and thus decreases inflammation. It is used for dry eye treatment, including meibomian gland dysfunction and inflammatory dry eye. Unfortunately, none of these agents are administrated orally.

### 6.2. RGD-Binding Integrins

A considerable effort is focused on drug development targeting eight (αIIbβ3, α5β1, α8β1, αvβ1, αvβ3, αvβ5, αvβ6, and αvβ8) RGD-binding integrins. Platelet-expressed integrin αIIbβ3 attracted a long-lasting interest for its role in cardiovascular and autoimmune diseases. Integrin αIIbβ3 also plays a role in cancer progression. Efforts in αIIbβ3 antagonist development led to the three already-mentioned drugs, abciximab, eptifibatide, and tirofiban, which have antithrombotic effects by blocking platelet aggregation. Other therapeutic agents against the αIIbβ3 integrin are under development, and more details can be found in selected reviews [24,152,231,325]. However, several oral αIIbβ3 antagonists failed in clinical trials, and, surprisingly, mortality was higher in treated patients than in placebo control [326]. The αIIbβ3 integrin is characteristic of an inactive conformation with low affinity for ligands in the resting platelet. The inside-out activation by binding talin and kindlin triggers a conformational change of integrins to a high affinity for ligands. The crystal structures of the αIIbβ3 integrin in complex with its inhibitors revealed that small inhibitors that failed in clinical trials stabilize high-affinity (open-extended) conformation [327]. The authors also found that water located in the MIDAS region stabilizes integrins in their low-affinity (bent) conformation, and drugs that stabilize this water also stabilize bent conformation. Recently, a small molecule Zalunfiban (RUC-4) (Figure 11d) integrin αIIbβ3 inhibitor that does not activate integrin has shown encouraging results [328]. Zalunfiban is now in Phase 3 clinical trial (CELEBRATE, ClinicalTrials.gov Identifier: NCT04825743, sponsored by CeleCor Therapeutics).

Drug discovery focused on the αv subfamily of RGD-binding integrins due to their association with cancer [268,325], arthritis [243], osteoporosis [329], age macular degeneration [26], and fibrotic diseases [246]. However, no drug was regulatory approved. Among five αv integrins, the αvβ3 integrin was the main focus for drug development for its implication in angiogenesis and tumor growth [322]. Recently, beyond αvβ3, αvβ1, αvβ5, and αvβ6 were also targeted. The most progressed αvβ3 inhibitor was cilengitide, a cyclic peptide developed by Merck. However, it failed in Phase III [330]. The development of many small molecules targeting αv inhibitors is in progress, and it is beyond the scope of this review to discuss all of them; details can be found in some reviews [14,25,321,322,331].

### 6.3. Leukocyte Integrins

Eight leukocyte integrins, αLβ2, αMβ2, αXβ2, αDβ2, αEβ7, α4β7, α9β1, and α4β1 play a vital role in inflammation and immunity. They are irreplaceable in leukocyte extravasation from blood to inflamed tissue [150,233]. Their inhibitors are potential therapeutics for modulating inflammation and are proven applicable in several diseases, such as IBD, psoriasis, dry eye diseases, asthma, multiple sclerosis, and cancer [24,231]. Three integrins, αLβ2, αEβ7, and α4β7, play an essential role in lymphocytes homing into the gut. Two mAb, natalizumab and vedolizumab (Figure 10), were successfully developed for treating UC and DC [239] and are already on the market. A human mAb AMG 181 was designed for IBD treatment by Amgen against the α4β7 integrin, now in Phase II trials. A small molecule inhibitor of the αLβ2 integrin is marketed to treat dry eye disease [26]. Another human mAb, etrolizumab, targets αEβ7 and α4β7 integrins by blocking interactions with their ligands E-cadherin and MAdCAM-1, respectively, and is now in Phase 3 clinical trial (BERGAMOT, ClinicalTrials.gov Identifier: NCT02394028, sponsored by Hoffmann-La Roche). Natalizumab is also used to treat chronic neurodegenerative disease of the central nervous system (CNS). However, in rare cases of treatment with natalizumab, a fatal multifocal encelophalopathy occurred. Various small molecules have been designed to tackle leukocyte integrins; details can be found in some reviews [25,27,231,239].

### 6.4. Integrin-Based Biomaterials for Bone and Tissue Repair

Recently, a new application of integrins emerged, namely in biomaterials applied in bone repair debilitated by non-healing skeletal defects caused by osteoarthritis, traumatic injury, and cancer [29,30,31,332,333,334]. Requirements for an orthopedic biomaterial stimulating implant integration in bone repair include the adhesion to osteoblasts and osteoprogenitor cells and support of their biological function. Interactions between bone cells (osteoblast and osteoprogenitor) and ECM regulate these processes. A group of integrins highly expressed in osteoblast and osteoprogenitor cells includes α1β1, α2β1, α3β1, α4β1, α5β1, α6β1, α8β1, α9β1, α4β7, αvβ3, and αvβ5 [30]. Therefore, much effort was focused on developing biomaterial functionalized by integrin ligands [332,334] and with good safety profiles [335].

Various strategies for synthesizing biomaterial functionalized with multivalent ECM-ligand were developed [29,31,332,333]. Though these technologies are in their infancy, some encouraging results have been obtained, such as biomaterial prepared from clinical-grade titanium and functionalized with various fractions of α5β1 binding fibronectin III being implanted into tibia defects in a rat model [336]. The results showed that targeting of the α5β1 integrin led to increased bone formation and bone repair in mice. Additionally, biomaterial with α4β1 ligands promotes bone formation and bone mass increase in mice. Interactions between bone cells and biomaterials are predominantly controlled by cell adhesion, which results from binding integrins to mimetics of their ECM ligands attached to biomaterial surface. The results suggest that applications of integrins’ functions to biomaterial have a potential not only in regenerative medicine but also in providing opportunity in device design and tissue remodeling. However, understanding several factors affecting the properties of biomaterials with a tuned integrin specificity and optimized ligand clustering remain to be resolved.

### 6.5. Molecular Modeling in the Design and Development of Integrin Antagonists

**Figure 12:**
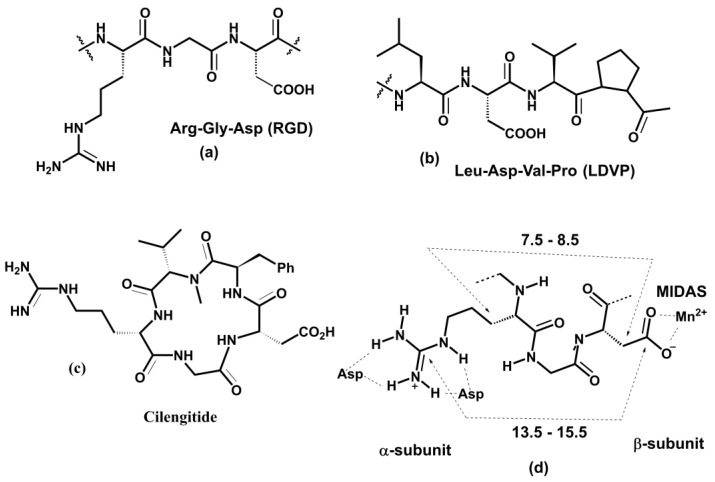
Schematic representation of (**a**) Arg-Gly-Asp (RGD), (**b**) Leu-Asp-Val-Pro (LDVP) integrin-binding motifs, (**c**) cyclic peptide RGD inhibitor cilengitide, and (**d**) key structural features of RGD-binding site.

Plethora molecules were synthesized as potential integrin antagonists [25,321,331,337,338,339]. In the beginning, their structures were mainly designed using chemical intuition to mimic integrin-binding moieties, such as RGD [340] or LDVP [229,341] (Figure 12a,b). Despite significant effort and many compounds entering clinical trials, only three small molecule inhibitors entered the market [14,24,231,320]. An integrin antagonist is a molecule that binds to the integrin natural ligand binding site (competitive antagonist) or another site (non-competitive antagonist) and thus blocks the integrin function. This term is analogous to competitive and non-competitive inhibition of enzymes. An agonist is a molecule that functions oppositely and initiates integrin function by binding to the receptor. Therefore, molecular modeling methods are suitable tools for rational developing antagonists. Recent progress in solving 3D structures of various integrins, either by X-ray crystallography, NMR, or homology modeling, provided the opportunity to use molecular modeling methods within rational drug discovery processes. Molecular docking, structure-based virtual screening, and molecular dynamics simulations are the most employed methods for this purpose. In light of several published reviews describing small molecule antagonists of integrins, our aim is not to review all available studies on the subject. Instead, we will discuss the impact of the structure-based drug design using selected results. Examples were chosen to include the antagonist discovery in relevant integrins, such as RGD-binding integrins and leukocyte integrins, and where molecular modeling methods played a prominent role. 

After identifying minimal integrin-binding motives, much effort was dedicated to developing their mimetics as potential integrin antagonists. Diverse strategies using various peptidic and non-peptidic scaffolds for preparing peptidomimetics were utilized. Cyclic peptidomimetics were often used as scaffolds to ensure a proper conformation of the binding moiety. For an effective rational drug design, the knowledge of the receptor 3D structure is essential. The solved X-ray structure of the αvβ3 complex with the cyclic antagonist cilengitide (Figure 12c, IC_50_ = 0.65 nM) provided information about the spatial arrangement of the RGD-binding motif [173]. 

Small molecule RGD antagonists bind to a binding pocket in a groove between the β-propeller of the α subunit and the βI domain of the β subunit atop the RGD-binding integrins such as αv, α5β1, α8β1, and αIIbβ3. The solved 3D structures of integrin-RGD ligand complexes revealed critical interactions in the binding site shown in Figure 12d [20,175,203,342]. Ligand binding to the β-subunit is stabilized using electrostatic interactions between a carboxylate group and the MIDAS bivalent metal ion. The guanidino group interacts with several negatively charged residues from the α-subunit. A typical distance between the carboxylate and guanidine groups is in the range of 13.5–15.5 Å and 7.5–8.5 Å between the β carbons of arginine and aspartate [338]. 

**Figure 13:**
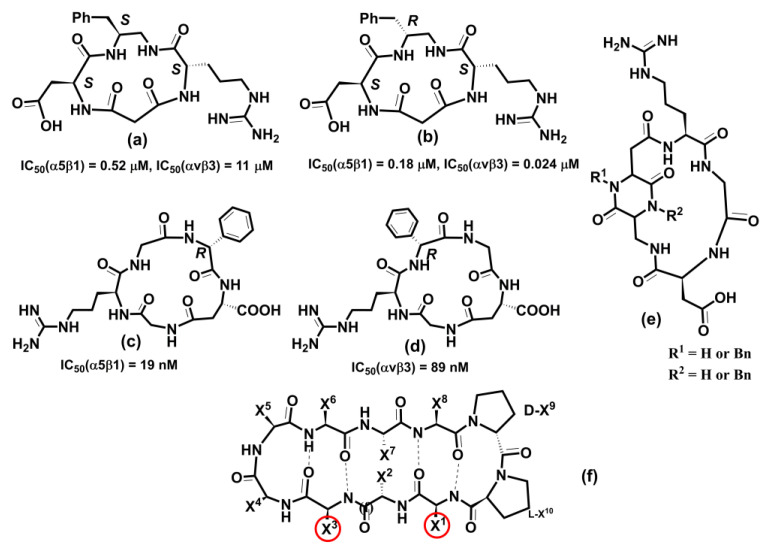
Schematic representation of RGD antagonists: (**a**) c[(*R*)-*β*-Phe-*ψ*(NHCO)Asp-*ψ*(NHCO)Gly-Arg]; (**b**) c[(*S*)-*β*-Phe-*ψ*-(NHCO)Asp-*ψ*(NHCO)Gly-Arg] [343]; (**c**) c[phg*iso*DGRG]; (**d**) c[G*iso*DGRphg] [344], where phg = D-phenylglycine; (**e**) *trans-cyclo*[DKP-RGD] [345]; and (**f**) 10-mer cyclic peptide [346], where X^1^-X^10^ represent amino acids side chains, and amino acids X^1^ and X^3^ are interacting with integrins.

The molecular modeling methods were also used to interpret different behavior of compounds c[(*R*)-*β*-Phe-*ψ*(NHCO)Asp-*ψ*(NHCO)Gly-Arg] and c[(*S*)-*β*-Phe-*ψ*-(NHCO)Asp-*ψ*(NHCO)Gly-Arg] (Figure 13a,b). These two compounds displayed different activities, though they have identical amino acid structures and differ only in the stereochemistry of the aromatic side chain. The S stereoisomer (Figure 13a) showed a submicromolar activity for α5β1 (IC_50_ = 0.52 μM) and two orders lower for αvβ3 (IC_50_ = 11 μM), while the R stereoisomer (Figure 13b) exhibited a potent dual antagonist activity, IC_50_ = 0.18 μM and IC_50_ = 0.024 μM for αvβ3 and α5β1, respectively. Solution conformations of both compounds determined by NMR spectroscopy and MD simulations were used as the starting structures for the following molecular docking into the αvβ3 integrin using Glide [343]. Molecular docking revealed that unfavorable interactions of the pseudoaxial orientation of the benzyl substituent in the S stereoisomer hinder a proper accommodation in the receptor site. In contrast, the pseudoequatorial orientation in the R-stereoisomer does not exhibit any steric hindrance.

Much attention has been paid to developing drugs for various diseases focusing on αv integrins, especially on αvβ3 integrin-involved cancer [264] and osteoporosis [329]. Molecular modeling methods were often included in the discovery process [131,196,346,347,348,349,350,351,352,353,354].

The αvβ3 crystal structure was used to analyze the binding mode of several potential inhibitors utilizing the docking approach [349]. The starting conformation of seven (four cyclic and three acyclic compounds) selected antagonists were based on solution conformations determined by NMR and MD simulations. Docking was performed using the AutoDock program with the backbone conformation held fixed while side chains were allowed to rotate. The obtained binding poses and scores compare well with experimental activity data. The results suggest that the orientation and distance between the positively-charged Arg and the negatively-charged carboxyl group of Asp groups influence the ligand binding. In addition, a pharmacophore model has been proposed for the rational design of αvβ3 ligands as potential anti-cancer drugs. The binding properties of several cyclic RGD antagonists were investigated by combining surface plasmon resonance (SPR) experiments and molecular docking using the LigandFit procedure [351]. SPR measurements identified cyclo[-Arg-Gly-Asp-ψ(triazole)-Gly-Lys] as the most active with K_D_ = 1.2 nM. Docking results showed that the binding of this cyclopeptide is consistent with the binding pose of cilengitide in αvβ3.

Cyclic peptides are relatively flexible molecules, and their conformations significantly influence pharmacological activity against integrins. Therefore, the reliable determination of their 3D molecular structure is essential for the design of potent and specific antagonists. In several studies, MD and MTD methods, NMR, and docking were used to determine the binding conformation of RGD-, DGR-, and isoDGR-containing cyclopeptides [131,344,345,348,355,356,357] (Figure 13).

The combination of MTD and docking simulations was employed to determine the binding conformation of cyclic peptides containing RGD-, DGR-, and isoDGR-moieties [131,348]. Conformational free energy surface (FES) as the function of two CVs, glycin dihedral angles φ and ψ, was calculated using well-tempered MTD for four cyclic peptides c(-RGDf(NMe)V-), DCGRC, CisoDGRC, and the N-terminal acetylated CisoDGRC (_ac_CisoDGRC). The MTD-calculated FES revealed that the conformational equilibria of these molecules are different. The docking of preferred conformations showed that c(-RGDf(NMe)V-), CisoDGRC, and _ac_CisoDGRC preferred conformations that fit inside the αvβ3 binding site, indicating that these extended conformations represent the bioactive conformation of ligands. MTD calculation also shows that the population of the dominant conformer is higher in _ac_CisoDGRC compared to CisoDGRC. The binding and competition experiments indicated that _ac_CisoDGRC has a stronger binding affinity than CisoDGRC [131]. The results suggested that the combination of MTD and docking provide the tool for distinguishing between binding and non-binding ligands and can be utilized for lead refinement in silico.

To decipher the selectivity of cyclo pentapeptides containing isoDGR motif (Figure 13c,d) against α5β1 and αv integrins, the 3D structure of various compounds was determined using NMR measurements, MD calculations, and docking studies [344,355]. The simulations confirmed the typical binding interactions and explained the selectivity of studied compounds.

Conformational analysis of the isoDGR cyclopeptides containing bifunctional DKP scaffolds (Figure 13e) was performed by combining the measurements NOESY NMR spectra and mixed Monte Carlo/stochastic dynamic [358] and the implicit water model [359]. The docking of two preferred conformers into the crystal structure of the αvβ3 integrin revealed meaningful ligand–αvβ3 interactions and identified the bioactive conformer.

Recently, the αvβ6 integrin has attracted research in treating the chronic lung disease idiopathic pulmonary fibrosis (IPF). MD simulations, free energy calculations, and docking were performed to investigate the binding properties of the αvβ6 integrin with its natural ligand and RGD mimetics. Calculations were carried out using a crystal structure of the αvβ6 integrin in complex with the pro-domain of its natural ligand, TGF-β1 (PDB code: 4UM9) [360]. The 1,8-naphthyridine moiety was used as a scaffold for potential antagonists. The results underlined hydrogen bond interactions between amino acids from αv and β6 subunits and ligands and electrostatic interactions between metal cation and ligands. The estimated binding affinities using FEP calculations were in reasonable agreement with the experiment, suggesting that many potential antagonists can be generated in silico, avoiding a time-consuming and expensive synthesizing investigation. Recently, a new de novo design algorithm was published [353], and the algorithm’s performance was tested by discovering potential antagonists of the αvβ6 integrin. From the chemical space of approximately 185,000 compounds, some novel molecules were suggested for synthesis as potential antagonists.

Infection by a foot-and-mouth disease virus commenced by attachment of the viral capsid to the host through RGD-containing αvβ6 integrin [361,362]. Molecular modeling methods were used to decipher the molecular basis of high affinity and specificity exhibited by a developed cyclic peptide [346]. The conformation of the 10-mer cyclic peptide (Figure 13f) was determined by NMR and docked into the active site of αvβ6 using MD simulations. The cyclic peptides have sub-nanomolar binding affinity against αvβ6 and showed promising results in bioimaging experiments on a human carcinoma cell line.

The integrin activation by an inhibitor [327] can be avoided by agents that bind integrin at sites other than the ligand-binding site. A molecular docking approach was used to discover such compounds [354]. The authors carried out a randomized docking of a mutated D1 domain of the CD2 protein (ProAgio) to a groove in the βI domain of the β3 subunit of the αvβ3 integrin with several different orientations. From 1000 generated conformers of the 1:1 complex of αvβ3:ProAgio, the structure with the lowest intermolecular interaction energy was selected for analysis. The estimated binding affinity, represented by a dissociation constant, was K_D_ = 4.3 nM. It was found that ProAgio binds only to αvβ3, and not to αIIbβ3, as a consequence of slight structural differences in the binding site of both integrins. ProAgio was found to induce apoptosis of cells and thus has therapeutic potential targeting integrin using the unique mechanism of action. ProAgio is in Phase 1 clinical trial (ClinicalTrials.gov Identifier: NCT05085548, sponsored by ProDa BioTech, LLC).

**Figure 14:**
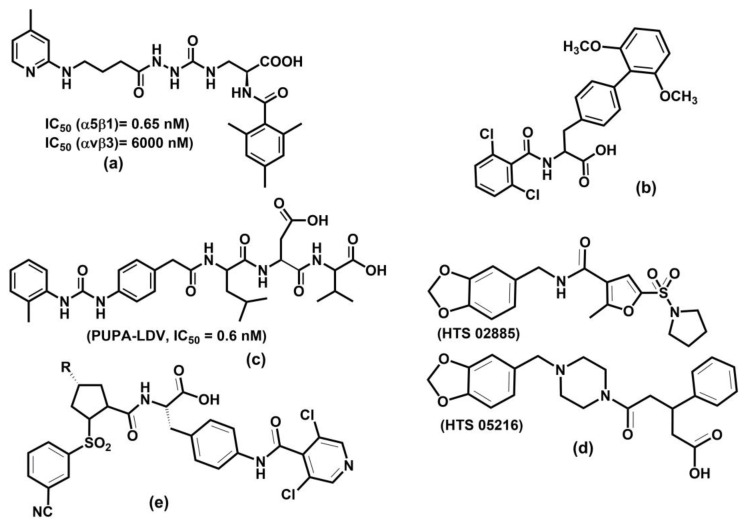
Schematic representation of (**a**) aza-glycine-based ligand [364], (**b**) phenylalanine derivative [365], (**c**) PUPA-LDV [366], (**d**) two virtual leads (HTS 02885 and HTS 05216) for development of VLA-4 antagonists [367] (**e**) MK-0617, R = 3,3-difluoropiperidine [368].

Integrin α5β1 is involved in age-related macula degeneration and cancer development. Integrin α5β1 and its ligand fibronectin play critical roles in angiogenesis [363]. Rational design procedures were used to discover and refine the structure of potent and specific α5β1 ligand [364]. Starting from a tyrosine scaffold, the approach utilizing SAR experiments and docking compounds into a developed homology model of α5β1 led to ligands based on an aza-glycine scaffold with affinities of ~1 nM and selectivity against αvβ3 that exceed 10^4^-fold (Figure 14a).

The leukocyte integrin α4β1 is associated with various diseases such as pulmonary fibrosis, multiple sclerosis, rheumatoid arthritis, asthma, COPD, and diabetes [369,370]. The α4β1 binds to fibronectin and VCAM-1 through minimum binding determinant LDV [341]. Targeting this integrin is challenging because no crystal structure was reported for α4β1. However, several molecular modeling studies focused on understanding binding interactions and developing specific and potent antagonists were published [365,366,367,371,372,373,374]. All these studies used homology models based on the crystal structure of the αvβ3 complex with cilengitide [146].

The structural basis for recognizing phenylalanine compounds (Figure 14b) by α4β1 was investigated using 128 antagonists [365]. A pharmacophore model was developed based on the VCAM-1 structure, then the pseudoreceptor model and 3D-QSAR were derived using electrostatic, hydrophobic, and hydrogen bond interactions. A docking experiment supported the reliability of the 3D-QSAR model, suggesting its application in the development of potential phenylalanine type of antagonists.

Antagonist binding modes of 4-[*N*’-(2-methylphenyl)ureido]phenylacetyl-Leu-Asp-Val (PUPA-LDV, Figure 14c) derivatives were investigated using several modeling methods, including docking, MD simulation, and free energy calculations [366]. The results revealed that the preferred conformation of PUPA-LDV in solution is similar to the one observed in the binding site of α4β1.

Pharmacophore modeling, virtual screening, and docking methods were employed to design potential lead compounds based on squaric acid, phenylalanine, and quinolinyl scaffolds [367]. The derived pharmacophore model from the training set of 110 diverse compounds was used for virtual screening commercial databases containing 110,000 diverse compounds, leading to two leads (Figure 14d) for developing α4β1 antagonists. The calculated GOLD score for both leads was higher than the GOLD score calculated for the best compound from the training set.

Several prolyl-N-isonicotinoyl-(L)-4-aminophenylalanine derivatives substituted at the proline 4-position with cyclic amines were prepared, and their activity screened against α4β1. The compounds with 3,3-difluoropiperidine at the proline 4-position, N-N-[(3-cyanobenzene) sulfonyl]-4(R)-(3,3-difluoropiperidin-1-yl)-(L)-prolyl-4-[(30,50-dichloro-isonicotinoyl) amino]-(L)-phenylalanine, (MK-0617) was the most potent derivative with IC_50_ = 0.03 nM. Moreover, MK-0617 exhibited good receptor occupancy. Interactions of the potent oral antagonist MK-0617 against α4β1 named (Figure 14e) were investigated with docking, MD simulations, and free energy calculations [372]. The analyses revealed that the studied antagonist binds in an extended conformation, and electrostatic interactions between the carboxyl group of ligand and MIDAS ion are crucial for the ligand potency. However, nonpolar and hydrogen bond interactions are also relevant for the proper orientation of antagonists in the integrin binding site. MK-0617 is in Phase 2b clinical trial (ClinicalTrials.gov Identifier: NCT05261126, sponsored by Merck Sharp & Dohme LLC).

The above-discussed molecules show that despite some drawbacks, the programs to develop integrin-targeting drugs continue. Currently, databases ClinicalTrials.gov and clinicaltrialsregister.eu list more than 120 clinical trials of integrin-based therapeutics. The selected molecules in recent clinical trials and reported data are listed in a recent review [24].

## 7. Summary and Perspectives

Integrins are cell adhesion and signaling glycoproteins with a large number of their receptors within the human body. They are involved in physiological and pathological processes, including tissue growth, inflammation, cancer, thrombosis, and autoimmune disorders. The association of integrins with various severe diseases caused an interest in developing agents that modulate integrin functions. The main focus has been on RGD-binding and leukocyte integrins. Despite significant efforts focused on integrin-based therapies, the results are unsatisfactory. The development of potent and specific small molecule inhibitors of integrin–ligand interactions is a challenging task. One of the challenges is that antagonists with sufficient potency in vitro often have poor pharmacokinetics. Another challenge is to develop specific antagonists. This challenge is inherent to the heterodimeric character of integrins, with the identical subunits being part of several different integrins. Therefore, a characteristic of integrin-based antagonists is often a lack of specificity. The last three decades documented the enormous progress in understanding integrins’ 3D structure, properties, and behavior and elucidated our knowledge of their biological functions and therapeutic potential. Recent improvement in molecular modeling methods makes them a powerful tool to provide valuable structural and energetic information on the integrin–ligand interactions at the atomic level and thus complement experimental data obtained by biochemical techniques. Therefore, a combination of new biology technologies, medicinal chemistry, and molecular modeling may provide new therapeutic agents with the required pharmacokinetics profile in a rational way.
